# Effect of Aerobic Exercise on Oxidative Stress and Inflammatory Response During Particulate Matter Exposure in Mouse Lungs

**DOI:** 10.3389/fphys.2021.773539

**Published:** 2022-02-03

**Authors:** Byunghun So, Jinhan Park, Junho Jang, Wonchung Lim, Saba Imdad, Chounghun Kang

**Affiliations:** ^1^Molecular Metabolism in Health and Disease, Exercise Physiology Laboratory, Inha University, Incheon, South Korea; ^2^Department of Sports Medicine, College of Health Science, Cheongju University, Cheongju, South Korea; ^3^Department of Biomedical Laboratory Science, College of Health Science, Cheongju University, Cheongju, South Korea; ^4^Department of Physical Education, College of Education, Inha University, Incheon, South Korea

**Keywords:** oxidative stress, particulate matter, *in vivo* mitophagy, mitochondria, pulmonary inflammation, treadmill exercise

## Abstract

Regular exercise provides several health benefits that can improve the cardiovascular and musculoskeletal systems, but clear evidence on the effect of exercise-induced hyperventilation in particulate matter (PM) exposure is still lacking. This study aimed to investigate the effects of exercise in PM exposure on reactive oxygen species (ROS) generation, inflammatory response, and mitochondrial integrity in human lung epithelial cells (A549), as well as in mouse lung tissue. In *in vitro* experiments, PM treatment was shown to significantly increased ROS production, and reduced cell viability and mitochondrial function in A549 cells. The mice were divided into four groups for an *in vivo* exercise experiment: control (CON), PM inhalation (PI), PM inhalation during exercise (PIE), and exercise (EX) groups. The PI and PIE groups were exposed to 100 μg/m^3^ of PM for 1 h per day for a week. The PIE and EX groups performed treadmill exercises every day for 1 h at 20 m/min for a week. The levels of pro-inflammatory markers (IL-6 and TNF-α) were significantly higher in the PI group than in the CON group (*P* < 0.001 and *P* < 0.01, respectively). The carbonyl protein level was decreased in EX vs. PI (*P* < 0.001). Mitochondrial fission (Drp1) content was significantly decreased in the EX vs. CON group (*P* < 0.01), but anti-mitochondrial fission (P-Drp1 Ser637) was increased in the EX vs. PI group (*P* < 0.05). Mitochondrial autophagy (mitophagy), which is an assessment of mitochondrial integrity, was markedly increased in PI vs. CON (*P* < 0.001), but the level was reversed in PIE (*P* < 0.05). Lung fibrosis was increased in PI vs. CON group (*P* < 0.001), however, the cells were rescued in the PIE (*P* < 0.001). The number of apoptotic cells was remarkably increased in the PI vs. CON group (*P* < 0.001), whereas the level was decreased in the PIE (*P* < 0.001). Taken together, these results showed that short-term exposure to PM triggers oxidative stress, pro-inflammatory responses, and apoptosis in the lungs, but the PM-induced adverse effects on the lung tissue are not exacerbated by exercise-induced PM hyperventilation but rather has a protective effect.

## Introduction

Particulate matter (PM) is recognized as a risk factor for various diseases and health problems. Exposure to PM is known to cause severe respiratory and cardiovascular diseases, including lung cancer (Raaschou-Nielsen et al., 2016), asthma (Lin et al., 2002), bronchitis, stroke, and atherosclerosis. Both systemic reviews and epidemiologic studies have suggested several pathological pathways that link PM exposure to cardiovascular diseases (CVDs): (1) pulmonary oxidative stress (Valavanidis et al., 2013), (2) systemic inflammation (Hoffmann et al., 2009), (3) perturbation of systemic autonomic nervous system balance (Rhoden et al., 2005), (4) potential translocation of PM into the systemic circulation (Brook et al., 2010), and (5) risk of premature mortality (Arriagada et al., 2019).

Exercise has been suggested to participate in preventing the risk of pathophysiological symptoms, including systemic inflammation and oxidative stress, through its anti-inflammatory and antioxidant effects (Benatti and Pedersen, 2015). Endurance exercise enhances the circulating levels of anti-inflammatory cytokines, such as interleukin-10 (IL-10) and interleukin-1 receptor antagonist (IL-1ra), and reduces pro-inflammatory conditions linked to type 2 diabetes, cardiovascular disease, and respiratory disease (Steensberg et al., 2003). Furthermore, exercise improves the antioxidant system in the body by increased transcription of genes coding for key antioxidant enzymes, such as superoxide dismutase, catalase, and glutathione peroxidase (Ji et al., 2016). These enzymes effectively eliminate ROS and reactive nitrogen species (RNS), but the imbalance between ROS/RNS generation and antioxidants makes the cells more vulnerable to oxidative damage (Ji, 2008). These redox signaling pathways can be regulated in response to endurance exercise adaptation through the Sestrin2-AMPK axis. It is well-known that adenosine 5-monophosphate (AMP)-activated protein kinase (AMPK) is a sensor of cellular energy that is activated under conditions of severe stress, such as an increase in AMP levels relative to ATP (Herzig and Shaw, 2018). Exercise, as a potent activator of AMPK, promotes mitochondrial biogenesis and anti-inflammatory responses by activating the downstream players of the peroxisome proliferator-activated receptor-γ co-activator 1α (PGC1α) (Jäger et al., 2007; Matsukawa et al., 2017). Recent studies have reported that endurance exercise increases the expression of Sestrin2 and can activate AMPK signaling by regulating the phosphorylation of AMPK at Thr172 (Budanov and Karin, 2008; Kim et al., 2020). Furthermore, activation of AMPK diminishes oxidative stress through the upregulation of nuclear factor erythroid-2 related factor 2 (Nrf2), a transcription factor that regulates antioxidant enzymes, such as glutathione S-transferase, peroxiredoxin, and sulfiredoxin (Suzuki et al., 2013; Joo et al., 2016). The role of endurance exercise in mitochondrial remodeling via redox signaling is well established. Mitochondria are dynamic organelles that continually undergo morphometric changes to induce fusion [mediated by mitofusin (Mfn) 1 and 2] and fission [mediated by dynamin-related protein 1 (Drp1)] to maintain mitochondrial quality control (Kang et al., 2009; Yan et al., 2012; Bohovych et al., 2015). Previous studies have shown that aerobic exercise training improves mitochondrial quality by regulating specialized autophagy pathways to selectively degrade defective mitochondria (Møller et al., 2015; Vainshtein et al., 2015; Laker et al., 2017). However, a recent report demonstrated that PM exposure impairs mitochondrial function, which is closely linked with the dysfunction of mitochondrial bioenergetics and ROS generation and causes systemic inflammation (Vitar et al., 2018; Magnani et al., 2020). Despite clear evidence of the role of exercise in mitochondrial quality control, there is no data that shows whether exercise-induced PM hyperventilation affects these pathways.

Epidemiological studies have shown that more particles are inhaled during exercise via hyperventilation. This can cause bronchial inflammation, leading to lung function impairment, which is characterized by decreased tidal volume and increased breathing frequency, depending on exercise intensity. However, despite the availability of well-documented studies on the adverse effects of PM exposure, there is limited understanding of the effects of exercise-induced PM hyperventilation on oxidative stress and inflammatory responses, due to the lack of relevant *in vivo* experimental models (Giles and Koehle, 2014; Curbani et al., 2019b). Moreover, most studies used the intranasal instillation protocol for PM exposure in an *in vivo* model; however, a higher concentration of PM was administered to murine models with this method compared to the physiological range of PM encountered daily (Curbani et al., 2019a).

Therefore, we first investigated the effect of 1-week, aerobic exercise on the oxidative stress and inflammatory response of mouse lung tissue during PM exposure using a specially designed treadmill system in the PM chamber, following *in vitro* experiments with human endothelial lung cells (A549 cells).

## Materials and Methods

### Cell Culture and Particulate Matter Treatment

A549 human lung adenocarcinoma basal epithelial cells were cultured in RPMI-1640 medium supplemented with L-glutamine (300 mg/L), HEPES (25 mM), NaHCO_3_ (25 mM), 10% fetal bovine serum, and 1% penicillin-streptomycin. Cultures were maintained at 37°C in a humidified atmosphere of 5% CO_2_ and were changed every 2 days. The stock solution of PM SRM 1648a Urban Particulate Matter (National Institute of Standards and Technology, United States) was prepared in dimethyl sulfoxide (DMSO) and then diluted to a working concentration of 1 mg/mL in phosphate buffer solution (PBS) using a vibrator for at least 20 min to avoid agglomeration of the suspended particles. The final concentration of DMSO was less than 0.1% per well. Cells were rinsed once with PBS prior to the addition of PM SRM 1648a.

### *In vitro* Transfection

*pMitoTimer* and *pMRX-IP-GFP-LC3-RFP* were obtained from AddGene. Briefly, A549 cells (3 × 10^5^ cells/well) were seeded into 6-well plates (SPL Life Sciences) and incubated overnight. The following day, cells were treated with different concentrations of SRM 1648a urban particulate matter suspension (5, 10, 20, and 40 μg/cm^2)^ and were transfected with either plasmid at 2 μg/well using polyethylenimine (PEI) and Opti-Mineral Essential Medium for 24 h incubation at 37°C and 5% CO_2_. All transfection experiments were performed in duplicate. The transfected cells were quantitated by fluorescence microscopy (Leica DMi8) for both green (excitation 420–460/emission 602–682 nm) and red (excitation 550–570/emission 602–682 nm) channels.

### Thiazolyl Blue Tetrazolium Bromide Assay

Thiazolyl Blue Tetrazolium Bromide assay was performed to measure cell viability. The cells were seeded at a density of 5 × 10^4^ cells/well in 96-well plates and incubated overnight. The cells were treated with different concentrations of SRM 1648a urban particulate matter suspension or normal culture medium for 6, 12, 24, and 48 h. After incubation with PM, 5 mg/mL MTT (Thiazolyl Blue Tetrazolium Bromide, GoldBio, T-030-5) solution (10 μL) was added to each well and the cell viability was measured by obtaining the absorbance at 570 nm.

### Internal Cellular Reactive Oxygen Species Assay

The internal cellular ROS in A529 cells were measured using the cell permeant reagent 2′,7′-dichlorofluorescin diacetate (DCFDA) based cellular ROS assay kit (#113851, Abcam, Cambridge, United Kingdom), according to the manufacturer’s instructions. The cells were treated with different concentrations of SRM 1648a urban particulate matter suspension or normal culture medium for 6, 12, 24, and 48 h. After incubation with PM, 100 μL diluted DCFDA was added to each well and incubated for 45 min at 37°C in the dark. The internal cellular ROS was measured using a fluorescence multi-well plate reader (Spectra max id3, Molecular Devices, United States) with excitation and emission wavelengths of 488 and 535 nm, respectively.

### Quantitative PCR Analysis

Total RNA was extracted from A549 cells using the RNA extract kit (NucleoSpin RNA Plus Kit, MACHEREY-NAGEL, Germany), according to the manufacturer’s instructions. Total RNA quantification and purity were estimated by NanoDrop (Spectra max id3, Molecular Devices, United States), and cDNA was synthesized using the iScript™ cDNA synthesis kit (Bio-Rad, United States). PTGS2, Il8, TNF, Ppargc1a, and IL1b levels were quantified using SYBR Green with a CFX96 real-time PCR System (Bio-Rad, United States). Forward and reverse primers for the aforementioned genes are provided in Table 1, and the relative mRNA levels were calculated using cycle threshold values, which were normalized to the internal control glyceraldehyde 3-phosphate dehydrogenase (GAPDH).

**TABLE 1:** Primer sequences for real-time PCR.

Target	Forward	Reverse
IL-8	5′-AAGAGAGCTCTGTC TGGACC-3′	5′-GATATTCTCTTGGCC CTTGG-3′
TNF-α	5′-GCCCATGTTGTAGCA AACCC-3′	5′-TATCTCTCAGCTCCA CGCCA-3′
PTGS2	5′-CCCGCCGCTGCGATGCT CGCCC-3′	5′-GACTTCTACAGTTCAGTC GAACG-3′

### The Extracellular Flux Cell Mitochondria Stress Analysis

The oxygen consumption rate (OCR) of cells was measured using a Seahorse XFp Analyzer (Agilent, United States), with the help of the Seahorse XFp Cell Mito Stress Test Kit, according to the manufacturer’s instructions. Briefly, 1.5 × 10^4^ A549 cells were seeded onto XFp cell culture miniplates and incubated in a CO_2_ incubator, the day before the experiment, and the probe plate was hydrated overnight 24-h prior to the assay. Cells were incubated for 1 h in XF base medium in non-CO_2_ incubation at 37°C and treated with 1.5 μM Oligomycin, 0.5 μM FCCP, and 0.5 μM Rotenone and antimycin A for analyzing OCR. The OCR was performed and analyzed after normalizing with the cell numbers using Seahorse Wave software and a seahorse report generator.

### Experimental Animals and Designs

Eight-week-old female FVB/N mice were randomly separated into four groups: CON, control group (*n* = 8); PI, PM exposure + sedentary group (*n* = 8); PIE, PM exposure + treadmill exercise group (*n* = 8); EX, treadmill exercise group (*n* = 8). To examine *in vivo* mitophagy, mt-Keima transgenic (heterozygous type (+/−) FVB/N) mice were kindly provided by Dr. Yoon, which were later bred and maintained in a specific pathogen-free facility. The mice were housed in a temperature (22°C) and humidity (40–60%) controlled environment with a 12-h light/12-h dark cycle and free access to allergen-free food and water. The protocol used in this study was approved by the Institutional Animal Care and Use Committee of the Institute (IACUC, approval number INHA 190211-616).

### Exercise and Particulate Matter Exposure

We designed a PM treadmill chamber to apply the inhalation protocol for exercise-induced hyperventilation during PM exposure (Windas, South Korea). The experimental apparatus consisted of a chamber, air circulator, PM supply, treadmill for the mouse, and a central control unit. PM was supplied to the treadmill chamber from the PM reservoir using a compressed air supply motor. The PM sample was obtained (ISO 12103-1) from POWDER TECHNOLOGY INC. and the distribution of PM size ranged from 0.97 to 22 μm. The AeroTrak 9306-V2 particle counter (TSI Incorporated, MN, United States) was used to measure and maintain a PM_2.5_ concentration of 100 μg/m^3^ in the chamber (Supplementary Figure 2).

The PIE and EX groups performed a forced treadmill exercise for 60 min at 20 m/min with a 5-degree uphill incline once a day for 1 week following 2 days of treadmill acclimatization. In order to ensure that the mice continue the treadmill exercises, air stimulation was applied in the tail direction when mice did not run, and electrical stimulation was avoided to reduce stress.

### Western Blot Analysis

The mice were sacrificed by injecting sodium pentobarbital (120 mg/kg), and a portion of the mouse lung was immediately frozen in liquid nitrogen. The frozen lungs were homogenized with a tissue grinder (Kinematica, United States) for 15 s in cold RIPA buffer (Cell Nest, South Korea), including a mixed protease inhibitor cocktail solution (GenDEPOT, South Korea). The total protein concentration was assayed using a Pierce BCA protein assay kit (Thermo Fisher Scientific, Waltham, MA, United States). Proteins were separated by SDS-PAGE and transferred to a PVDF membrane (Amersham, Germany) and blocked with 5% skimmed milk or bovine serum albumin prior to primary antibody incubation. The primary antibodies used in this assay are listed in Table 2. The membranes were developed, following secondary antibody incubation, by using Pierce™ ECL western blotting substrate (Thermo Fisher Scientific, Waltham, MA, United States) and analyzed using ImageJ software (version 1.8.0_172; National Institutes of Health, Bethesda, MD, United States).

**TABLE 2:** List of primary antibodies.

Antibody	Source	Clonality	Manufacturer (cat. no.)	Dilution
Anti-Bax	Mouse	Monoclonal	SANTA CRUZE (#sc20067)	1:2,000
Anti-Sestrin2	Rabbit	Polyclonal	Proteintech (#10795-1-AP)	1:2,000
Anti-NFκB	Rabbit	Monoclonal	Cell Signaling (#8242)	1:2,000
Anti-p62	Mouse	Monoclonal	ABCAM (#ab56416)	1:2,000
Anti-LC3A/B	Rabbit	Polyclonal	Cell Signaling (#4108)	1:2,000
Anti-AMPKα	Rabbit	Polyclonal	Cell Signaling (# 2532)	1:2,000
Anti-pAMPKα Thr172	Rabbit	Monoclonal	Cell Signaling (#2535)	1:2,000
Anti-PGC-1α	Mouse	Monoclonal	SANTA CRUZE (#sc-517380)	1:2,000
Anti-ATG13	Rabbit	Monoclonal	Cell Signaling (#13468)	1:2,000
Anti-ATG101	Rabbit	Monoclonal	Cell Signaling (#13492)	1:2,000
Anti-Parkin 2	Mouse	Monoclonal	SANTA CRUZE (#sc-32282)	1:2,000
Anti-AKT	Rabbit	Monoclonal	Cell Signaling (#4691)	1:2,000
Anti-BID	Mouse	Monoclonal	SANTA CRUZE (#sc-373939)	1:2,000
Anti-Bcl2	Mouse	Monoclonal	SANTA CRUZE (#sc-7382)	1:2,000
Anti-COX2	Mouse	Polyclonal	Cayman (#aa 584-598)	1:2,000
Anti-Drp1	Rabbit	Monoclonal	Cell Signaling (#8570)	1:2,000
Anti-pDrp1 S616	Rabbit	Polyclonal	Cell Signaling (#3455)	1:2,000
Anti-pDrp S637	Rabbit	Polyclonal	Cell Signaling (#4867)	1:2,000
Anti-OPA1	Rabbit	Polyclonal	ABCAM (#ab42364)	1:2,000
Anti-β-actin	Mouse	Monoclonal	SANTA CRUZE (#sc-47778)	1:2,000

### *In vivo* Mitophagy Image Analysis

To measure *in vivo* mitophagy, we used mt-Keima transgenic mice, which allow the detection of mitophagy with a mitochondria-targeting sequence from COX VIII, that can be easily targeted to the mitochondrial matrix along with the coral-derived pH-dependent fluorescent protein Keima (Katayama et al., 2011). This model can illustrate the physiological status of the mitochondria by displaying a green color under physiological conditions (pH 8.0), which turns red upon acidic conditions inside the lysosomes (pH 4.5) (Sun et al., 2015). To observe *in vivo* mitophagy, the lung tissue was washed with cold PBS and 1.0 μm thick sections were excised using the Brain Slicer Matrix (Sun et al., 2017). The tissue sections were transferred to a confocal dish (SPL), and the nuclei were stained with Hoechst 33342 and 4,6-diamidino-2-phenylindole (DAPI) solution (5 μg/mL) for 5 min on ice (Thermo Fisher Scientific). Fluorescence intensities were measured using a laser confocal microscope (LSM 510 META, ZEISS), adjusted to excitation of 488 and 561 nm for green and red, respectively, with a single emission wavelength of 620 nm. ImageJ software was used for statistical analysis, and mitophagy changes were evaluated by comparing the red/green ratio.

### Enzyme-Linked Immunosorbent Assay

The plasma expression levels of the pro-inflammatory cytokines TNF-α and interleukin-6 (IL-6) were measured by Quantikine™ ELISA immunoassay (Quantikine™ ELISA Mouse TNF-α and IL-6 Immunoassay; R&D System, Inc., United States). ELISA was performed according to the manufacturer’s instructions.

### Morphological Analysis of Tissues

Lung tissues were fixed with 4% paraformaldehyde in PBS overnight and then processed for paraffin embedding. The paraffin blocks were sectioned to be 5 μm thick and stained with hematoxylin and eosin (H&E) and Masson’s trichrome (MT). Images of the sections were captured using a Leica DM i8 microscope (Leica, Germany) and quantified using the ImageJ analysis program (NIH, Bethesda, MD, United States) by measuring the relative area of fibrosis (fibrosis area/total area × 100) by MT staining.

### Terminal Deoxynucleotidyl Transferase dUTP Nick End Labeling Assay

DNA fragmentation was detected using an ApopTag peroxidase in situ apoptosis detection kit (Millipore, Bedford, MA, United States) according to the manufacturer’s instructions. TUNEL-positive cells were detected and counted in multiple sections of the lung using a fluorescence microscope.

### Statistical Analysis

Experimental data were expressed as the mean ± standard error of the mean (SEM), and group comparisons were made by one-way ANOVA and Tukey-Kramer *post hoc* test with a standard significance threshold (*P* < 0.05).

## Results

### Particulate Matter Induces Oxidative Stress and Mitochondrial Dysfunction in A549 Cells

**FIGURE 1:**
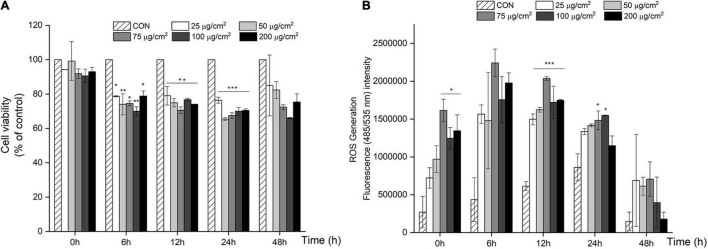
PM-induced negative effects on cell viability and over-production of ROS in A549 cells. **(A)** Cell viability decreased in a dose and time dependent manner in A549 cells after 24 and 48 h of PM treatment. **(B)** ROS generation increased with PM treatment in A549 cells after 24 and 48 h of incubation. Values are the ratio and the means ± SEM. *P < 0.05, **P < 0.01, and ***P < 0.001 compared with control.

We first screened for PM-induced changes in cell viability using the MTT assay. After 6 h, cell viability significantly decreased by 21–34% (*P* < 0.05; Figure 1A) when treated with a PM concentration ranging from 25 to 200 μg/cm^2^ (equivalent to 119–950 μg/mL), and similar results were obtained after 12, 24, and 48 h.

To further assess the effect of PM treatment on oxidative stress, ROS production was measured using a DCFH-DA probe in A549 cells after treatment with PM. As shown in Figure 1B, there was a significant increase in ROS in response to PM treatment in a dose-dependent manner starting from 25 μg/cm^2^ to 75 μg/cm^2^ vs. CON (*P* < 0.001; Figure 1B), after 12 h of PM exposure. In contrast, ROS production rapidly decreased in A549 cells after a 48 h treatment with PM.

### Particulate Matter Exposure Leads to Mitochondrial Integrity and Autophagy Activity in A549 Cells

**FIGURE 2:**
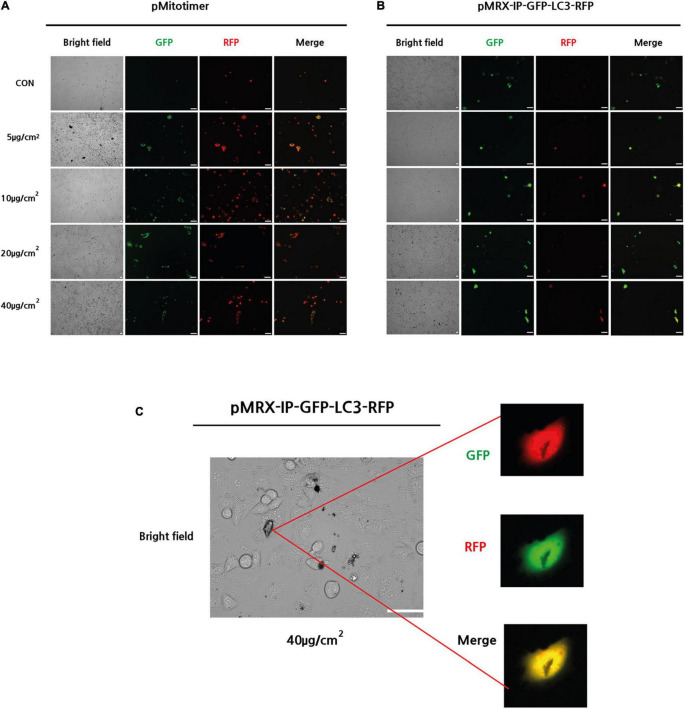
Exposure to PM-induced mitochondrial damage and autophagy in A549 cells. **(A)** Representative images of *MitoTimer* fluorescence spectrum shift in response to PM induce mitochondrial oxidation. **(B,C)** Representative images of *pMRX-IP-GFP-LC3-RFP* transfected cells during PM-induced autophagy. (Scale bar = 50 μm).

To evaluate mitochondrial integrity after PM treatment, *pMitotimer* were transfected into A549 cells and incubated for 24 h. *pMitotimer* is a useful tool designed to assess mitochondrial content, structure, and damage under oxidative stress. A fluorescent *Timer* protein encoded in the cytochrome *c* oxidase subunit VIII gene can indicate green fluorescence in newly generated mitochondria and shift to red when mitochondria have low membrane potential (Laker et al., 2014). After 24 h of exposure to PM, the *pMitotimer-transfected* A549 cells showed a PM-induced dose-dependent shift toward red puncta, which are oxidized mitochondria in the cells (Figure 2A).

Next, *pMRX-IP-GFP-LC3-RFP* was transfected into A549 cells to monitor autophagy activity after PM exposure with 24 h incubation. The red signal for autolysosomes and a yellow signal indicating autophagosomes in the cytosol were observed in response to treatment with PM at a dose of 40 μg/cm^2^ (Figure 2B). Moreover, PM particles were shown to closely adhere to the cell surface, overlapping transfected autophagy parts (Figure 2C). Therefore, it can be suggested that PM exposure induces increased mitochondrial oxidation (dehydrogenization) and autophagy in A549 cells.

### Particulate Matter Increases the Expression of AMPK and Sestrin2 Levels *in vitro*

**FIGURE 3:**
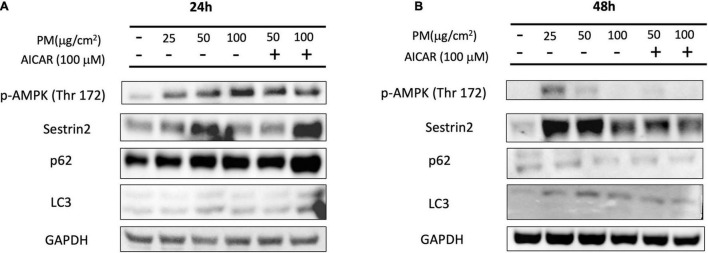
Effect of PM exposure and AICAR on AMPK-Sestrin2 protein expression in A549 cells. **(A)** Representative western blot images of p-AMPK (Thr 172), Sestrin2, p62, and LC3 at **(A)** 24 h and **(B)** 48 h of PM exposure and AICAR treatment.

After showing that PM causes enhanced mitochondrial oxidation and autophagy, we measured the changes in the protein levels of the relevant pathways to examine how PM induces ROS generation. Figure 3A shows that 24 h of PM exposure in A549 cells resulted in a significant increase in p-AMPKα (phosphorylation at threonine; Thr-172) protein levels in a dose-dependent manner. Sestrin2 protein expression was elevated in response to PM exposure of 25 μg/cm^2^ and 50 μg/cm^2^ at the 24 h time point. However, these proteins showed reduced expression after 48 h of exposure to PM (Figure 3B). Next, we treated A549 cells with AICAR (100 μM), which mimics the effect of exercise, to determine whether upregulation of AMPK activity can increase autophagy after PM exposure. Sestrin2 content was elevated after 24 h due to both PM exposure as well as AICAR treatment with PM, but protein content showed a slight decline after AICAR treatment with PM after 48 h. p62 and LC3 II levels were elevated due to treatment of AICAR with PM after 24 h exposure, whereas p62 and LC3 II protein levels were reduced after 48 h of PM exposure, despite treatment with AICAR.

**FIGURE 4:**
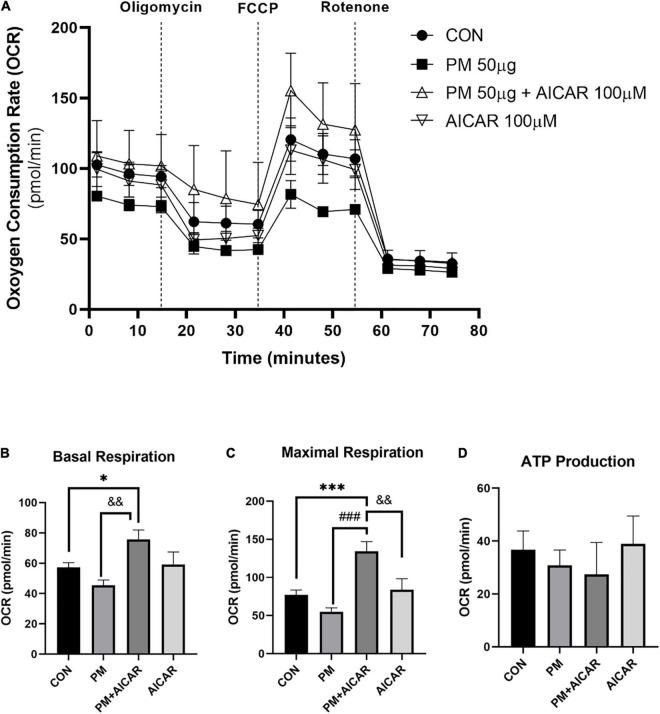
Oxygen consumption rate (OCR) during PM exposure and AICAR treatment in A549 cells. **(A)** A549 cells were treated with oligomycin, FCCP, and rotenone/antimycin A to measure the effect of mitochondrial stress on OCR, upon PM exposure and AICAR treatment at 24 h incubation. The mitochondrial stress test was used to measure the bioenergetic parameters, **(B)** basal respiration, **(C)** maximal respiration, **(D)** adenosine triphosphate (ATP) production. Values are the means ± SEM. *P < 0.05 vs. CON, ^***^P < 0.001 vs. CON, ^###^*P* < 0.001 vs. PM, ^&⁣&^*P* < 0.01 vs. PM+AICAR.

Moreover, we observed the effect of PM exposure on mitochondrial respiration *in vitro* using the XFp seahorse system (Figure 4A). There was a lower level of basal respiration in the PM group, but this level was increased in PM+AICAR (*P* < 0.01; Figure 4B). Furthermore, maximal respiration was restored by 2.5-fold in PM+AICAR vs. PM alone (*P* < 0.001; Figure 4C). However, there was no difference in ATP production between groups (Figure 4D).

### Particulate Matter Induces Inflammatory Response in Human Alveolar Basal Epithelial Cells

**FIGURE 5:**
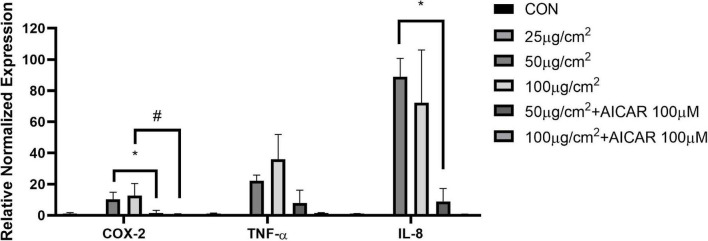
AICAR reduced the expression of PM-triggered inflammatory factors in A549 cells. mRNA expression of pro-inflammatory factors (COX-2, TNF-α, and IL-8) with and without AICAR treatment during PM exposure for 48 h in A549 cells. Values are mean ± SEM. *P < 0.05 vs. 50 μg/cm^2^, ^#^*P* < 0.05 vs. 100 μg/cm^2^.

To examine how PM exposure affects pro-inflammatory cytokines, we screened for changes in mRNA levels in response to 48 h of PM treatment in A549 cells. The mRNA expression levels of cyclooxygenase-2 (COX-2), IL-8, and TNF-α were elevated by PM treatment, however, the effects were reversed in COX-2 and IL-8 by AICAR treatment (*P* < 0.05, respectively) (Figure 5).

### Exercise Can Ameliorate Particulate Matter-Induced Oxidative Stress in *in vivo* Model

**FIGURE 6:**
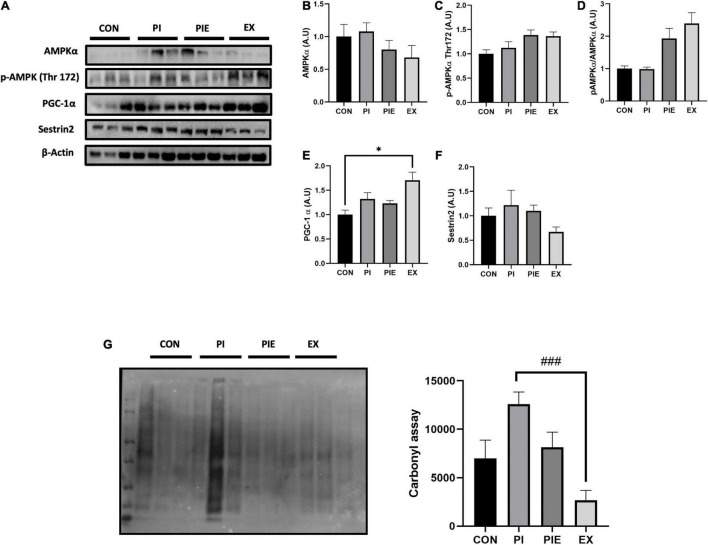
Exercise ameliorated PM-induced oxidative stress in the mouse lungs. **(A)** Representative western blot images of AMPKα, p-APMKα Thr 172, PGC-1α, and Sestrin2. Quantification of western blot analysis was performed to access the effect of PM exposure and exercise on AMPKα **(B)**, p-APMKα Thr 172 **(C)**, p-AMPKα Thr 172/AMPKα ratio **(D)**, PGC-1α **(E)**, Sestrin2 **(F)**. Carbonyl assay **(G)** and it’s quantification. Values are the means ± SEM (*n* = 6). *P < 0.05 vs. CON, ^###^*P* < 0.001 vs. PI.

There was no difference in AMPKα protein and AMPKα Thr 172 phosphorylation levels between groups in mice lung tissues (Figures 6A–D). Moreover, the ratio of p-AMPKα to AMPKα had a tendency to increase in PIE and EX groups, but no changes were observed in the other groups. PGC-1α level was increased in EX vs. CON (*P* < 0.05) (Figure 6E). Sestrin2 protein content was not changed between groups (Figure 6F). To assess whether PM or exercise affects oxidative stress, carbonyl protein levels were measured in mice lung tissue. The carbonyl protein level was modestly increased in the PI group vs. the CON group, but no significance was observed among groups, except for EX group where the level was markedly decreased by 4.72-fold vs. PI (*P* < 0.001; Figure 6G).

### Exercise Can Ameliorate Particulate Matter-Induced Inflammatory Response in *in vivo* Model

**FIGURE 7:**
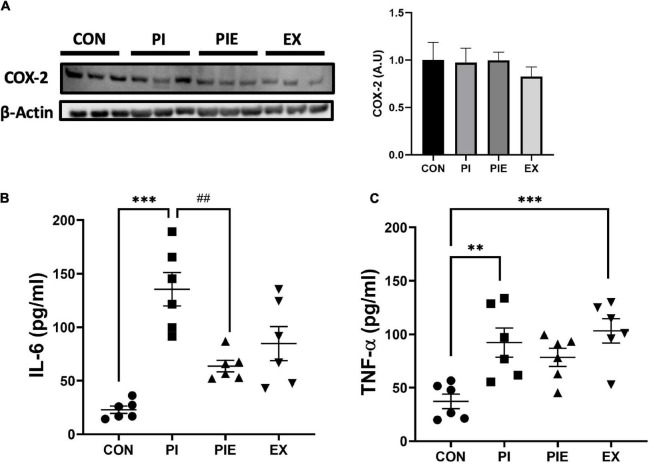
Effect of PM and exercise on the expression of pro-inflammatory markers. **(A)** Representative western blot image of COX2 in the lung. Effect of 1 week of PM exposure and/or exercise on expression of IL-6 **(B)** and TNF-α **(C)** in the plasma. Levels were determined by ELISA assay. Values are the means ± SEM (*n* = 6). ^**^P < 0.01 vs. CON, ^***^P < 0.001 vs. CON, ^##^*P* < 0.01 vs. PI.

To assess the effect of PM exposure on pro-inflammatory markers, IL-6 and TNF-α levels were determined in mouse plasma by ELISA. IL-6 levels were significantly higher in the PI group (*P* < 0.001) than in the CON group. Interestingly, the PIE group showed decreased expression of IL-6 compared to the PI group (*P* < 0.01). In addition, TNF-α levels were elevated in PI (*P* < 0.01) and EX (*P* < 0.001) vs. the CON group, and no change was observed in the PIE group. there was no change in COX-2 expression among the groups (Figure 7).

**FIGURE 8:**
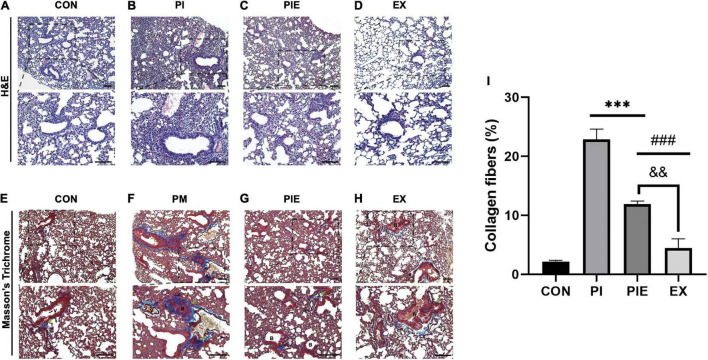
Histochemical and biochemical analyses of the lung fibrosis. **(A–D)** Hematoxylin and eosin straining of the lung tissue sections. **(E–H)** Masson’s trichrome (MT) staining of the lung tissue sections, with collagen fibers are shown in blue. Green arrows indicate collagen fibers. **(I)** Quantification of the collagen fibers observed in MT-stained lung tissue sections. B, bronchiolar; V, pulmonary capillary. Values are the means ± SEM (*n* = 3). ^***^P < 0.001 vs. CON, ^###^*P* < 0.001 vs. PI, ^&&^*P* < 0.05 vs. PIE.

We evaluated lung inflammation by H&E staining and lung fibrosis by MT staining Figures 8A–H. MT staining showed that exposure to PM significantly increased lung fibrosis by 21% in the PI (*P* < 0.001) and 10% in the PIE (*P* < 0.001) vs. CON group. The ratio of collagen fibers were markedly decreased by 11% in the PIE vs. PI group (*P* < 0.001), whereas EX group showed the lowest percentage of collagen fibers similar to CON (Figure 8I).

### Particulate Matter Leads to Changes in Mitochondrial Dynamics Related Proteins in *in vivo* Model

**FIGURE 9:**
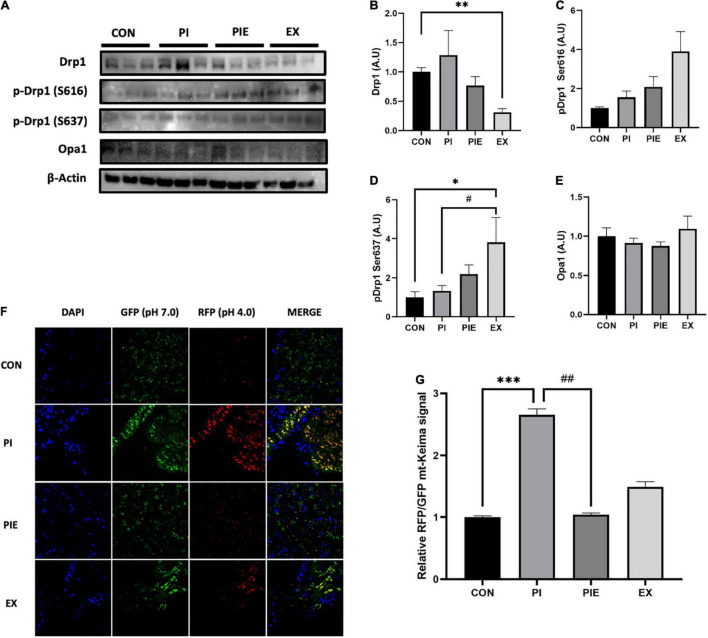
PM exposure increased mitochondrial fission markers and *in vivo* mitophagy signals in the mt-Keima mice lung. **(A)** Representative western blot images of Drp1, p-Drp1 Ser616, p-Drp1 Ser637, and Opa1. Western blot analysis was performed to access the effect of PM exposure and exercise on Drp1 **(B)**, p-Drp1 Ser616 **(C)**, p-Drp1 Ser637 **(D)**, and Opa1 **(E)**. **(F)** Representative *in vivo* mitophagy in the lung of mt-Keima mice. **(G)** Assessment of the RFP/GFP ratio for mitophagy signals. Scale bar = 50 μm. Values are the means ± SEM (*n* = 6). *P < 0.05 vs. CON, ^**^P < 0.01 vs. CON, ^***^P < 0.001 vs. CON, ^#^*P* < 0.05 vs. PI, ^##^*P* < 0.01 vs. PI.

Exercise significantly decreased the protein expression of mitochondrial dynamic marker, Drp1 which is required for mitochondrial fission (*P* < 0.01). However, phosphorylation of Drp1 at serine-637, which inhibits mitochondrial fission, was significantly increased in the EX group compared to that in the CON (*P* < 0.05) and PI group (*P* < 0.05). There was no change in the phosphorylation level of Drp1 at serine-616, which is known to promote mitochondrial fission activity, between the CON and PI group (Figures 9A–E). To examine *in vivo* fluorescence images of mitophagy, mt-Keima signals were imaged using a confocal microscope. The RFP/GFP ratio was markedly increased by 2.5-fold in the PI vs. CON groups (*P* < 0.001), but the level was decreased by 2.5-fold in the PIE vs. PI groups (*P* < 0.01) (Figures 9F,G).

### Particulate Matter Leads to the Activation of Autophagy in *in vivo* Model

**FIGURE 10:**
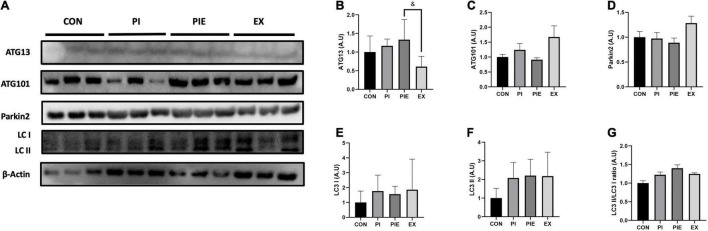
PM exposure increased the expression of autophagy-related markers in the lung. **(A)** Representative western blot images of ATG13, ATG101, LC3 I, II, and Parkin 2. Western blot analysis was performed to access the effect of PM exposure on ATG13 **(B)**, ATG101 **(C)**, Parkin 2 **(D)**, LC3 I **(E)**, LC3 II **(F)**, and LC3 II/I ratio **(G)**. Values are the means ± SEM (*n* = 6). ^&^*P* < 0.05 vs. PIE.

The expression of autophagy-related protein 13 (ATG13), which is known to be required for autophagosome formation, was 2.2-fold higher in the PIE group (*P* < 0.05) than in the EX group (Figures 10A,B). There was no significant difference between the groups for ATG101 and Parkin 2 expression (Figures 10C,D). Figures 10E–G showed that PM exposure caused no significant changes in LC3 I and LC3 II protein levels between groups.

### Particulate Matter Leads to Apoptosis-Related Protein Activation in an *in vivo* Model

**FIGURE 11:**
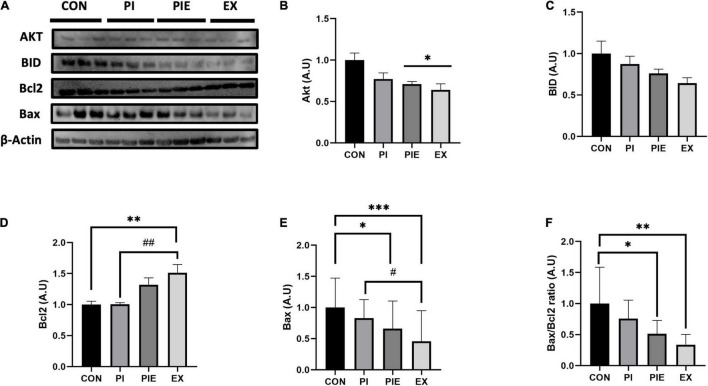
Exercise can ameliorate apoptosis-related markers during PM exposure and exercise in the lung. **(A)** Representative western blot images of Akt, BID, and Bcl2. Western blot analysis was performed to assess the effect of PM exposure on Akt **(B)**, BID **(C)**, Bcl2 **(D)**, Bax **(E)**, and Bax/Bcl2 ratio **(F)**. Values are the means ± SEM (*n* = 6). *P < 0.05 vs. CON, ^**^P < 0.01 vs. CON, ^***^P < 0.001 vs. CON, ^#^*P* < 0.05 vs. PI, ^##^*P* < 0.01 vs. PI.

**FIGURE 12:**
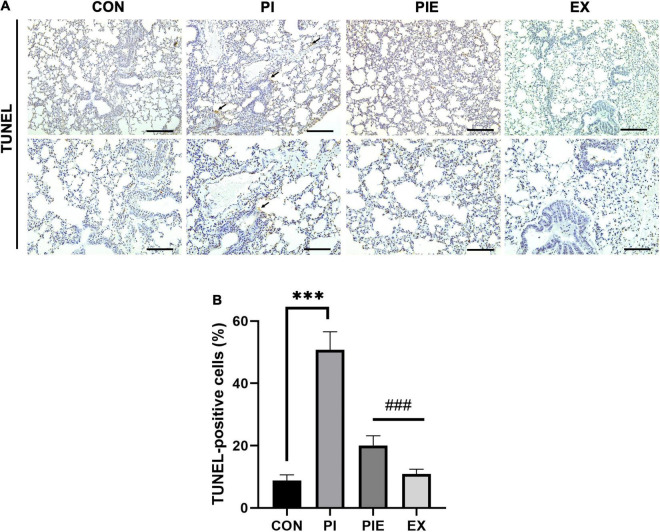
Apoptosis in the lung assessed by TUNEL assay. **(A)** Representative TUNEL staining in lung. Black arrow indicates TUNEL-positive cell. **(B)** Percentage of TUNEL-positive cells. Values are the means ± SEM (*n* = 3). ***P < 0.001 vs. CON, ^###^*P* < 0.001 vs. PI.

Next, we measured the levels of apoptosis-related proteins by western blotting (Figure 11). Bcl2, which is a regulator of anti-apoptotic signals on the outer mitochondrial membrane, was 1.5-fold higher in the EX group than in the PI group (*P* < 0.01), which was similar to the CON. The level of Bid, a pro-apoptotic protein, was not changed between groups, but Bax was significantly decreased in the EX vs. CON group (*P* < 0.001). In addition, Bax content was also significantly decreased in the EX vs. PI group (*P* < 0.05) and the PEI vs. CON group (*P* < 0.05). There was no significant change in the ratio of Bax/Bcl2 between CON and PI group. Bax/Bcl2 ratio was decreased by 48 and 66% in the PIE (*P* < 0.05) and the EX (*P* < 0.01) vs. CON group, respectively, but the level in the PIE was still 17% higher than EX group. To detect cells undergoing apoptosis, a TUNEL assay was performed to quantitate the apoptotic cells in the lung (Figure 12A). TUNEL staining showed that positive cells were significantly increased by 42% in the PI vs. CON group (*P* < 0.001), but the level was markedly decreased by 31% in the PIE vs. PI groups (*P* < 0.001). In addition, TUNEL-positive cells were markedly decreased by 30% in the PIE vs. PI group (*P* < 0.001). EX showed the lowest number of TUNEL positive cells (Figure 12B).

## Discussion

This study aimed to determine how exercise in a high-concentration PM environment could affect oxidative stress, inflammatory responses, and apoptosis in the lung. The PM metabolic chamber was designed to study exercise-induced hyperventilation through treadmill exercise in a natural environment, mimicking a real setting of high concentrations of PM exposure. During 1 week of exercise-induced upon PM exposure, exercise reduced the toxic effects of PM in pulmonary tissue, including oxidative stress, apoptosis, and pro-inflammatory response. Moreover, exposure to PM during exercise may inhibit PM-induced adverse effects, including systemic inflammation and apoptosis. Several studies have investigated the mechanism of PM-induced changes using *in vitro* models to understand the effects of PM on the body (Han and Zhuang, 2020; Liu et al., 2020; Wang et al., 2020). However, there is a limitation in reproducing a realistic representation of PM exposure *in vivo*. Most *in vivo* studies have shown the use of intra-tracheal/nasal installations that are used to mimic PM, but in this case, usually, a higher concentration of PM than the natural atmospheric concentration is introduced (Jarmuszkiewicz et al., 2020). Therefore, in this study, we investigated the effect of exercise-induced hyperventilation on the respiratory system to mimic the real environment of PM exposure in the chamber for *in vivo* studies.

To investigate the adverse effects of PM exposure, we screened A549 cells after PM treatment. Our data showed that treatment with PM markedly reduced cell viability and elevated ROS generation in A549 cells. In addition, PM treatment increased mitochondrial oxidation and autophagy. To mimic the exercise stimulus, we treated A549 cells with AICAR, which restored mitochondrial OCR. In addition, decreased inflammatory markers were observed in PM-exposed A549 cells after AICAR treatment. The *in vitro* outcomes suggest that PM treatment might be closely linked with the regulation of ROS generation and inflammatory markers; however, treatment with AICAR can alleviate PM-induced abnormal mitochondrial functions and inflammatory marker expression. Acute inflammation, which is a local defense response to damage as well as an adaptive response, within a short time plays an important role in tissue regeneration. However, long-term persistent inflammatory responses are more detrimental (Zhou et al., 2016; Chen et al., 2018). Exposure to PM triggers an inflammatory response at the site of injury, such as infiltration of macrophages and neutrophils in lung tissue which leads to systemic inflammation (Miyata and van Eeden, 2011; Xu et al., 2013; Costa et al., 2017).

Exercise has been known to reduce the systemic inflammatory response, and research on the effect of exercise in a fine dust environment is being actively studied. A recent study showed that a low-level exposure to PM_2.5_, during exercise, promoted heart oxidative stress and increased the levels of pro-inflammatory proteins in mice (Mai et al., 2017). In another study, 20 volunteer students participated in a field Cooper test, and were assessed for their fitness in high levels of PM. They showed increased lactate levels, decreased red blood cell counts, and hemoglobin, than the group exposed to lower levels of PM (Kargarfard et al., 2011). Our data showed that pro-inflammatory factors such as IL-6 and TNF-α were significantly increased in the PI group compared to the CON group, but exercise ameliorated its expression. Similar results from previous studies and current studies suggest that aerobic exercise has a protective role in decreasing the inflammatory response in the lungs (Gonçalves et al., 2012; Vieira et al., 2012; Cardoso et al., 2018; Lee et al., 2019). Recent studies have shown that exposure to PM affects respiratory frequency, the number of breaths, and breathing volume (Brown et al., 2013). In addition, the chemical composition of PM induces oxidative stress and inflammatory responses, which can negatively affect the pulmonary system. When starting exercise, minute ventilation (VE) increases 3–4 fold by light exercise compared to resting status, but high-intensity exercise could cause a 6–10 fold increase in VE (Daigle et al., 2003; Oravisjärvi et al., 2011). This means that there is a possibility of increased susceptibility to respiratory disease due to PM exposure, with increased exercise intensity (Giles and Koehle, 2014). Despite the benefits of exercise, such as diminishing oxidative stress and reducing the inflammatory response, there is limited understanding of the benefits or adverse effects of exercise-induced hyperventilation in PM exposure. PM consists of metal and carbon components, including iron (Fe), copper (Cu), and zinc (Zn), which can trigger adverse effects linked to oxidative stress conditions in the tissues (Chen and Lippmann, 2009). Some studies have demonstrated that exposure to PM is one of the mediators of oxidative stress that increases pro-inflammatory responses and biomarkers, such as lipid or protein oxidation products (Pardo et al., 2015; Tseng et al., 2017; Wang et al., 2017; Yuan et al., 2019). Our data demonstrated an increase in ROS levels after treatment with PM using the DCFH-DA probe in A549 cells. In addition, carbonyl protein content was higher in the PI group than in the CON group (*P* < 0.05). These data suggest that PM can trigger ROS production, resulting in mitochondrial dysfunction due to oxidative stress, but exercise can reduce the inflammatory response in the lungs.

There is evidence that the Sestrin2-AMPK axis pathway is redox-sensitive (Lee et al., 2013). Sestrins are regulators of Nrf2, an antioxidant transcription factor, that suppresses oxidative damage and eliminates damaged mitochondria by autophagy (Bae et al., 2013). Sestrins have three paralogs: Sestrins1-3, which are important for the regulation of ROS found in different tissues (Lee et al., 2013), including the lungs (Heidler et al., 2013), kidneys (Hamatani et al., 2014), and embryonic fibroblasts (Peng et al., 2014). Specifically, Sestrin1 and Sestrin2 can reduce ROS levels in *in vitro* models (Budanov et al., 2004). The antioxidant function of Sestrin2 promotes the activation of AMPKα (Budanov and Karin, 2008). A recent study showed that knockdown of Sesn2 can markedly increase the ROS level, mitochondrial damage, and cell pryroptosis in a mouse macrophage cell line, J774.A1. In addition, in the lipopolysaccharide (LPS)-induced acute lung injury model, the level of inflammatory markers IL-1 beta and IL-18 were increased in serum and bronchoalveolar lavage fluid (BALF) in the Sesn2 knockout mice (Sesn^–^/^–^) (Wu et al., 2021). These results suggest that Sestrin2 can be protected the LPS-induced lung injury through Sestrin2-mediated mitophagy. Our data showed that treatment with AICAR, an AMPK activator, with PM was sufficient to increase the level of Sestrin2 and the LC3II/I ratio in A549 cells. However, there was no difference in Sestrin2 *in vivo* levels between any groups, and Sestrin2 had a tendency to be decreased in the EX group. In addition, AMPKα and p-AMPKα Thr 172 protein contents were not changed among groups. Recently, a study showed that 4 weeks of treadmill exercise in mice resulted in a decreased the levels of Sestrin2 and p-AMPKα expression in the quadriceps muscle (Crisol et al., 2018). Although there was no increase in Sestrin2 and AMPKα expression due to exercise, exercise might be counteracted to increase ROS levels in the PM exposure environment. However, few studies have attempted to identify the mechanisms regulating ROS due to exposure to PM.

Mitochondria are essential organelles that continuously undergo remodeling by fission and fusion to preserve mitochondrial quality control, such as mitophagy (Tilokani et al., 2018). Defective or damaged mitochondria can be removed by mitophagy, which is a special autophagic pathway through lysosomal activity (Pickles et al., 2018). Dynamin-related protein 1 (Drp1) is a key regulator of mitochondrial fission and is recruited to the outer membrane of mitochondria in response to oxidative stress (Lackner and Nunnari, 2009). Excessive fission can induce morphological fragmentation to disconnect mitochondrial dynamics (Dagda et al., 2009). It has been well-documented that the abnormal mitochondria and impaired removal of damaged mitochondria are associated with the onset of several pathological lung diseases, including chronic obstructive pulmonary disease (COPD) and pulmonary fibrosis (Cloonan and Choi, 2016). Specifically, Liu et al. (2020) reported that PM exposure for 24 h increase the levels of mitochondrial fission markers (Drp1 and P-Drp1 Ser616) and decreased mitochondrial respiratory function in human lung epithelial cells (BEAS-2B) (Liu et al., 2020). Our data showed that 1 week of PM exposure and exercise can change mitochondrial fission proteins in the lungs. In addition, exercise significantly reversed the PM-induced mitophagy *in vivo*. These results suggest that exercise might inhibit excessive mitochondrial fragmentation and delete damaged mitochondria by mitophagy during PM exposure. It has proved challenging to monitor mitophagy in mammalian tissues, because mitochondria are dynamic organelles that morphologically change by fission and fusion. To overcome some of the technical limitations to assess *in vivo* mitophagic flux during a variety of physiological conditions, a transgenic mouse model which is mt-Keima model was developed to measure the quantification of mitophagy *in vivo*. The mt-Keima model can be detected by a pH-dependent fluorescence based Keima protein, which is targeted to the mitochondrial matrix (Sun et al., 2017; Philp et al., 2021). Under the physiological condition, mt-Keima can be excited by shorter-wavelength of 400 nm (pH 8), whereas a longer-wavelength of 568 nm is required for excitation in acidic conditions (pH 4.5), when the mitochondria is within the acidic lysosomes during mitophagy (Sun et al., 2015, 2017). Recently, it was observed that exhaustive treadmill exercise for 3 days led to an elevation of the mitochondrial lysosome intensity and area in the heart muscle using mt-Keima mice model (Liu et al., 2021). However, there are few studies to measure exercise-induced *in vivo* mitophagy using mt-Keima model.

Particulate matter exposure was associated with the accumulation of defective mitochondria at the cellular level, provoking oxidative stress and apoptosis. A previous study showed that PM exposure triggered endothelial cell apoptosis through increased ER stress by overloading intracellular Ca^2+^ and oxidative stress (Wang et al., 2020). Another study reported an increased inflammatory response, damaged mitochondria, and apoptosis in response to PM exposure for 24 h in Sprague Dawley (SD) rats (Li et al., 2021). Our data showed an overexpression of Bcl2 and a reduction in the expression of BID protein in comparison to CON in mice lungs. In addition, 1 week of exercise under PM exposure inhibited apoptosis in the PIE group compared to the PI group in the lung with TUNEL staining. Taken together, these results indicate that even short-term exposure to PM can be vulnerable to increased pro-inflammatory responses, apoptosis, and oxidative stress generation in the lung, which may be linked to the occurrence of damaged mitochondria in the lung. However, exercise can ameliorate PM-induced oxidative stress and pro-inflammatory responses despite elevated hyperventilation during exposure to high PM concentrations.

The limitation of this study was that exercise-induced hyperventilation was assumed by increasing exercise intensity and respiration frequency, but we did not know how much exercise-induced-hyperventilation occurred in the mice lungs during 1 week of PM exposure. In conclusion, we found that PM exposure increases oxidative stress, pro-inflammation, mitochondrial fission, and apoptosis in the lung, but exercise can ameliorate these adverse effects by using 1 week PM with the exercise model in a specially designed chamber. This study lays the foundation for future work on the long-term PM exposure model to determine the effect of exercise-induced hyperventilation during PM exposure.

## Data Availability Statement

The original contributions presented in the study are included in the article/Supplementary Material, further inquiries can be directed to the corresponding author/s.

## Ethics Statement

The protocol used in this study was approved by the Institutional Animal Care and Use Committee of the Institute in Inha University (IACUC, approval number INHA 190211-616).

## Author Contributions

BS and JP contributed equally to this work. BS, JP, and CK conceptualized the study. BS wrote the original draft. BS, JP, WL, SI, and CK reviewed and edited the manuscript prior to submission. All authors contributed and approved the final manuscript.

## Conflict of Interest

The authors declare that the research was conducted in the absence of any commercial or financial relationships that could be construed as a potential conflict of interest.

## Publisher’s Note

All claims expressed in this article are solely those of the authors and do not necessarily represent those of their affiliated organizations, or those of the publisher, the editors and the reviewers. Any product that may be evaluated in this article, or claim that may be made by its manufacturer, is not guaranteed or endorsed by the publisher.
