# Supplementary material for: Effect of Aerobic Exercise on Oxidative Stress and Inflammatory Response During Particulate Matter Exposure in Mouse Lungs
Source: Front Physiol. 2022 Feb 3;12:773539. doi: 10.3389/fphys.2021.773539 (PMC8850364; doi:10.3389/fphys.2021.773539)
Supplement: Supplementary file 1 [file Presentation_1.PPTX]

## Slide 1
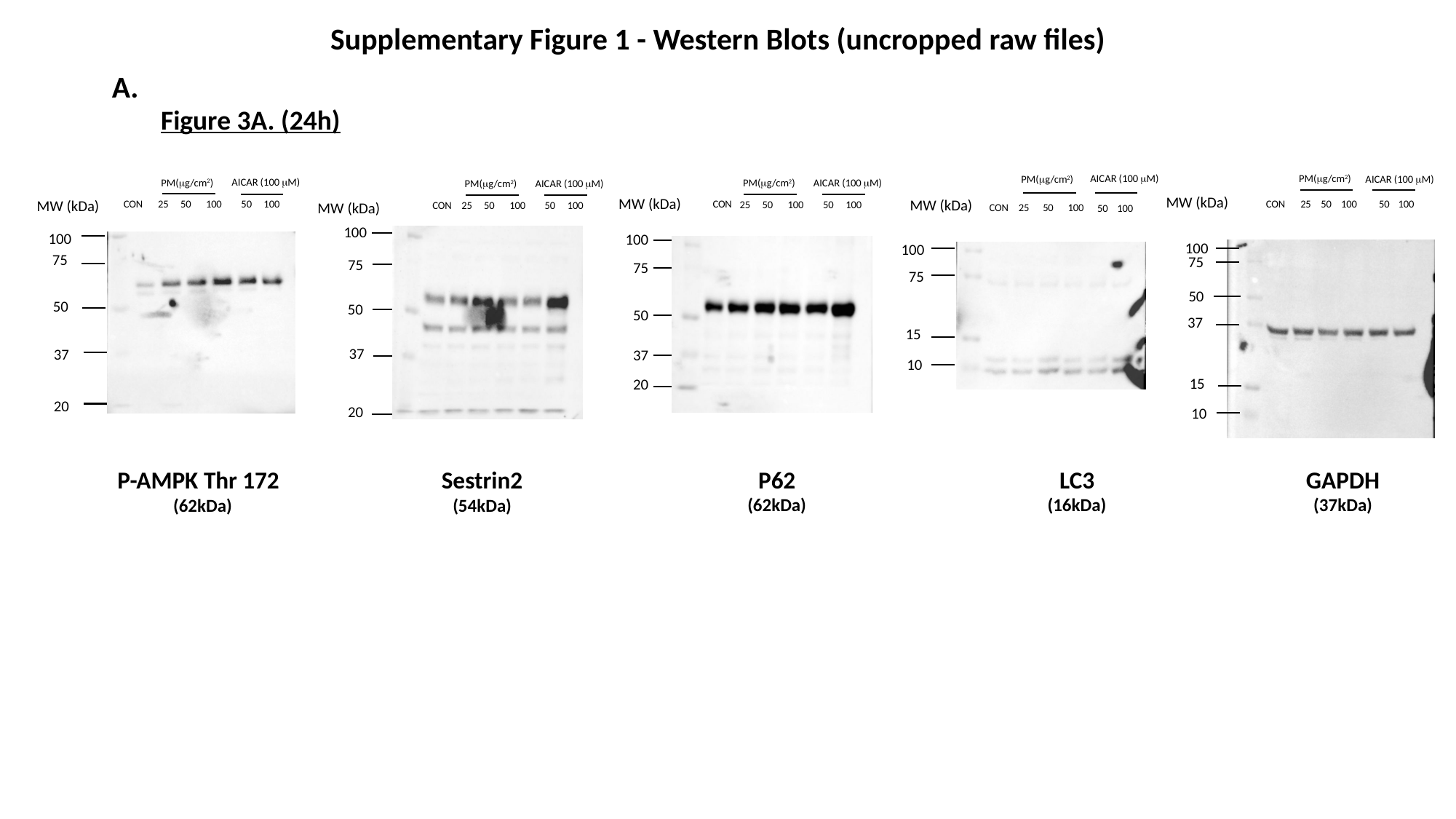

Supplementary Figure 1 - Western Blots (uncropped raw files)
A.
Figure 3A. (24h)
PM(μg/cm2)
AICAR (100 μM)
AICAR (100 μM)
PM(μg/cm2)
AICAR (100 μM)
PM(μg/cm2)
AICAR (100 μM)
PM(μg/cm2)
AICAR (100 μM)
PM(μg/cm2)
MW (kDa)
MW (kDa)
MW (kDa)
MW (kDa)
CON
CON
25
50
100
50
100
CON
25
50
100
50
100
25
50
100
50
100
25
50
100
50
100
MW (kDa)
CON
CON
25
50
100
100
50
100
75
50
37
20
100
75
50
37
20
100
75
50
37
20
100
75
50
37
15
10
100
75
15
10
P62
(62kDa)
LC3
(16kDa)
GAPDH
(37kDa)
P-AMPK Thr 172
(62kDa)
Sestrin2
(54kDa)
35
20

## Slide 2
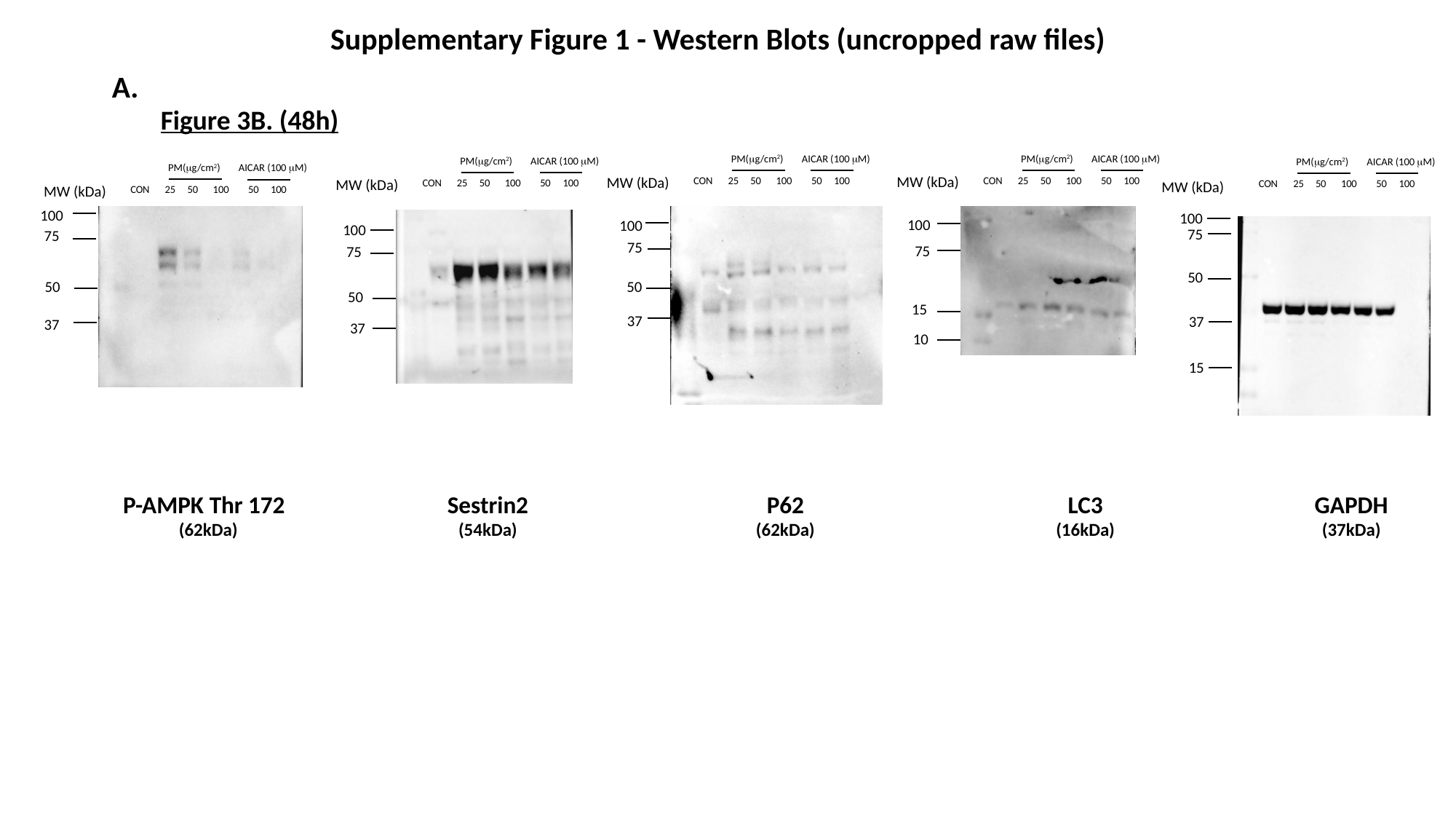

Supplementary Figure 1 - Western Blots (uncropped raw files)
A.
Figure 3B. (48h)
AICAR (100 μM)
PM(μg/cm2)
AICAR (100 μM)
PM(μg/cm2)
AICAR (100 μM)
PM(μg/cm2)
AICAR (100 μM)
PM(μg/cm2)
AICAR (100 μM)
PM(μg/cm2)
MW (kDa)
MW (kDa)
CON
25
50
100
50
100
CON
25
50
100
50
100
MW (kDa)
CON
25
50
100
50
100
CON
25
50
100
50
100
MW (kDa)
MW (kDa)
CON
25
50
100
50
100
100
75
50
37
100
75
50
37
15
100
75
15
10
100
75
50
37
100
75
50
37
P-AMPK Thr 172
(62kDa)
Sestrin2
(54kDa)
P62
(62kDa)
LC3
(16kDa)
GAPDH
(37kDa)

## Slide 3
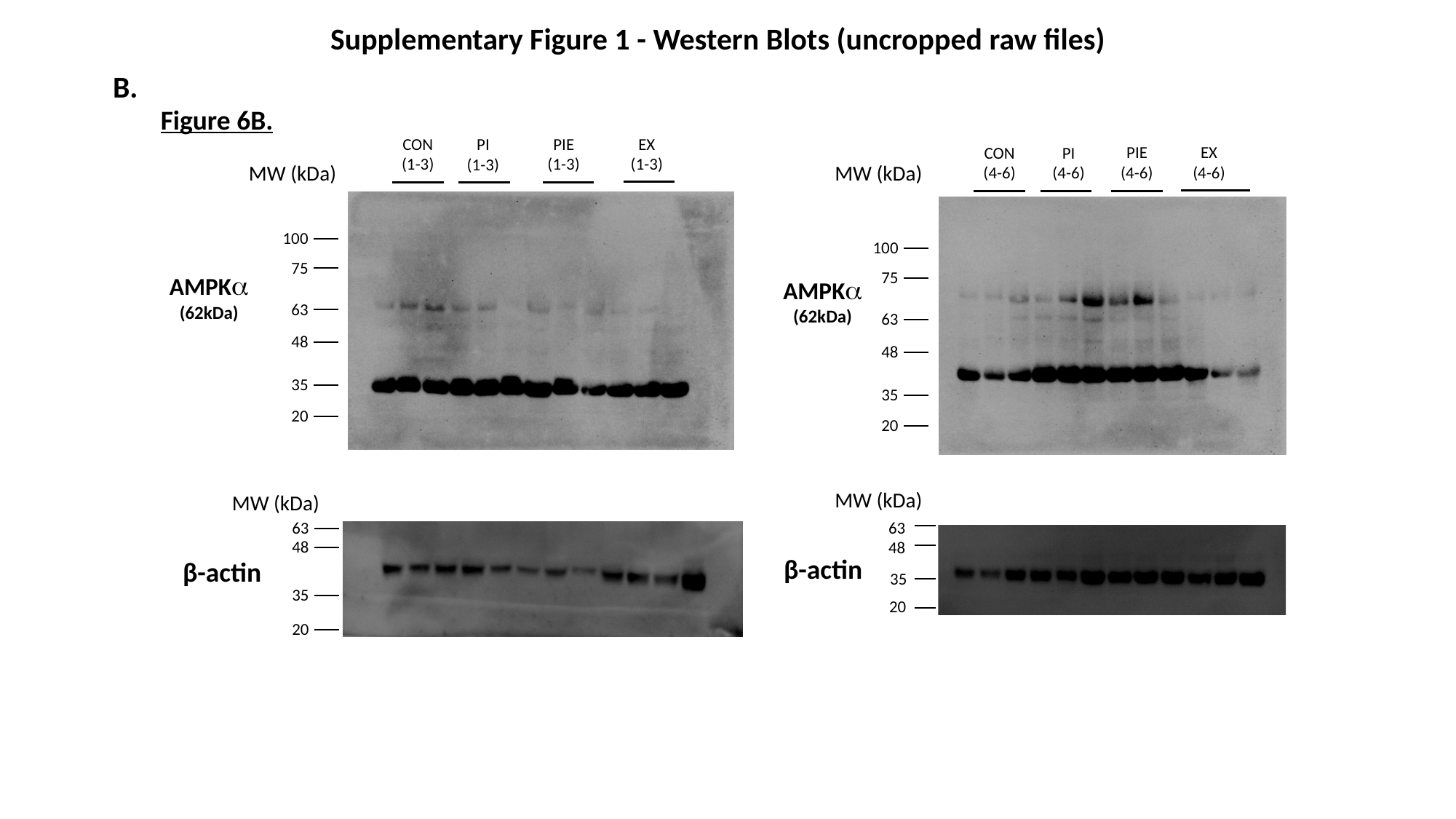

Supplementary Figure 1 - Western Blots (uncropped raw files)
B.
Figure 6B.
EX
(1-3)
PIE
(1-3)
CON
(1-3)
PI
(1-3)
PIE
(4-6)
EX
(4-6)
PI
(4-6)
CON
(4-6)
MW (kDa)
MW (kDa)
100
100
75
75
AMPKα
(62kDa)
AMPKα
(62kDa)
63
63
48
48
35
35
20
20
MW (kDa)
MW (kDa)
63
63
48
35
20
48
β-actin
β-actin
35
20

## Slide 4
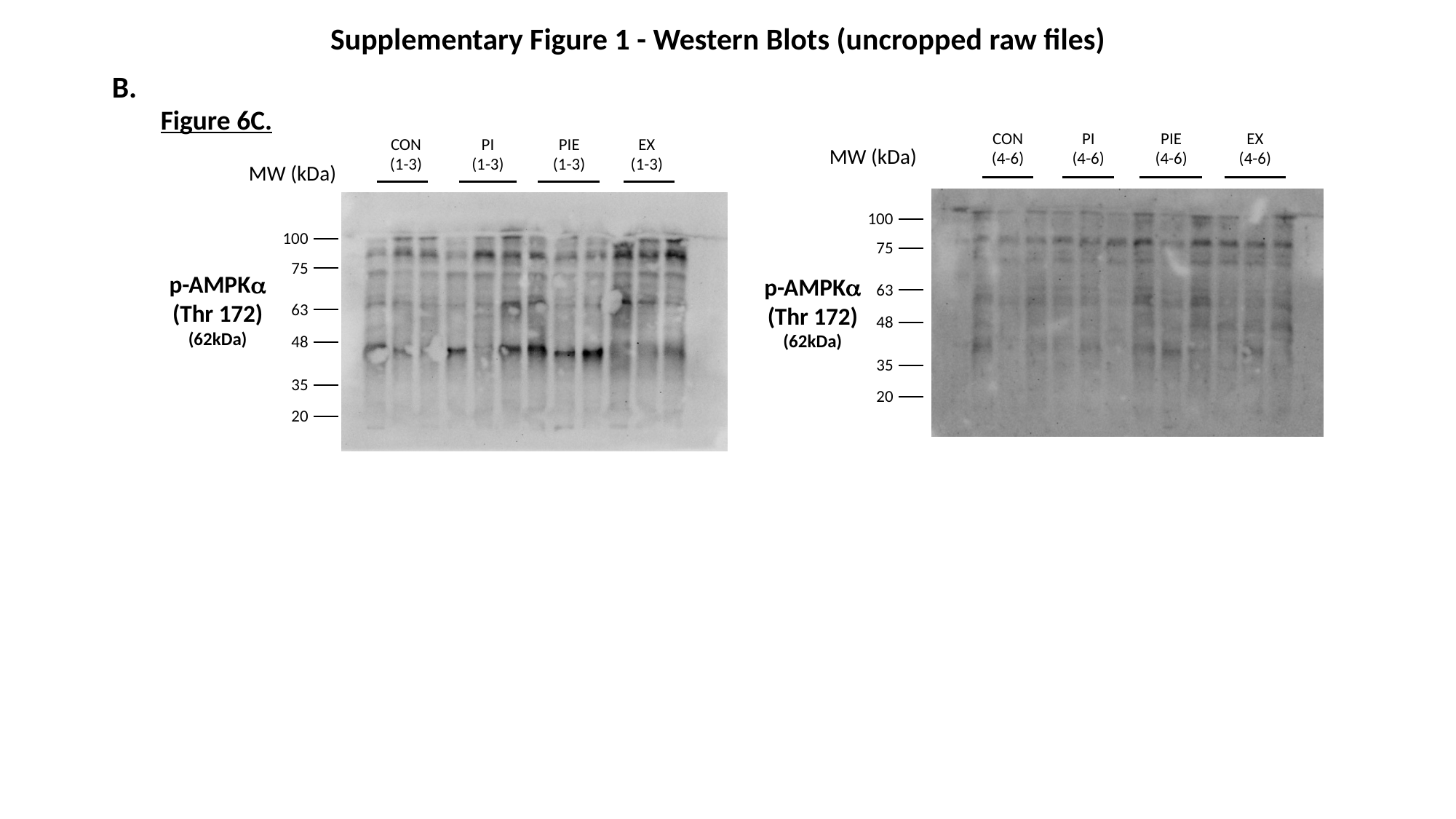

Supplementary Figure 1 - Western Blots (uncropped raw files)
B.
Figure 6C.
CON
(4-6)
PI
(4-6)
PIE
(4-6)
EX
(4-6)
CON
(1-3)
PI
(1-3)
PIE
(1-3)
EX
(1-3)
MW (kDa)
MW (kDa)
100
75
63
48
35
20
100
75
63
48
35
20
p-AMPKα (Thr 172)
(62kDa)
p-AMPKα (Thr 172)
(62kDa)

## Slide 5
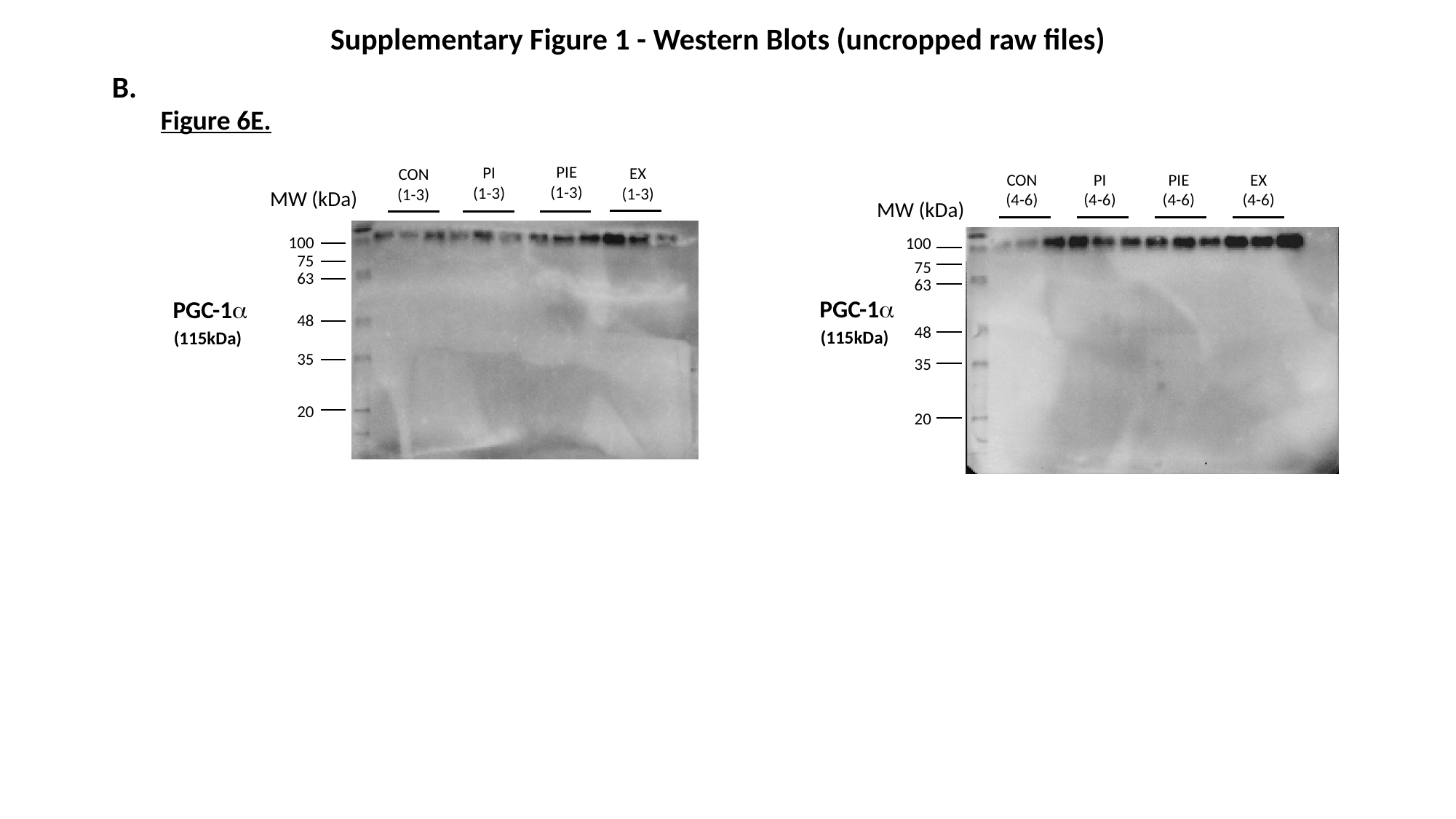

Supplementary Figure 1 - Western Blots (uncropped raw files)
B.
Figure 6E.
PIE
(1-3)
PI
(1-3)
EX
(1-3)
CON
(1-3)
CON
(4-6)
PI
(4-6)
PIE
(4-6)
EX
(4-6)
MW (kDa)
MW (kDa)
100
100
75
75
63
63
PGC-1α
(115kDa)
PGC-1α
(115kDa)
48
48
35
35
20
20

## Slide 6
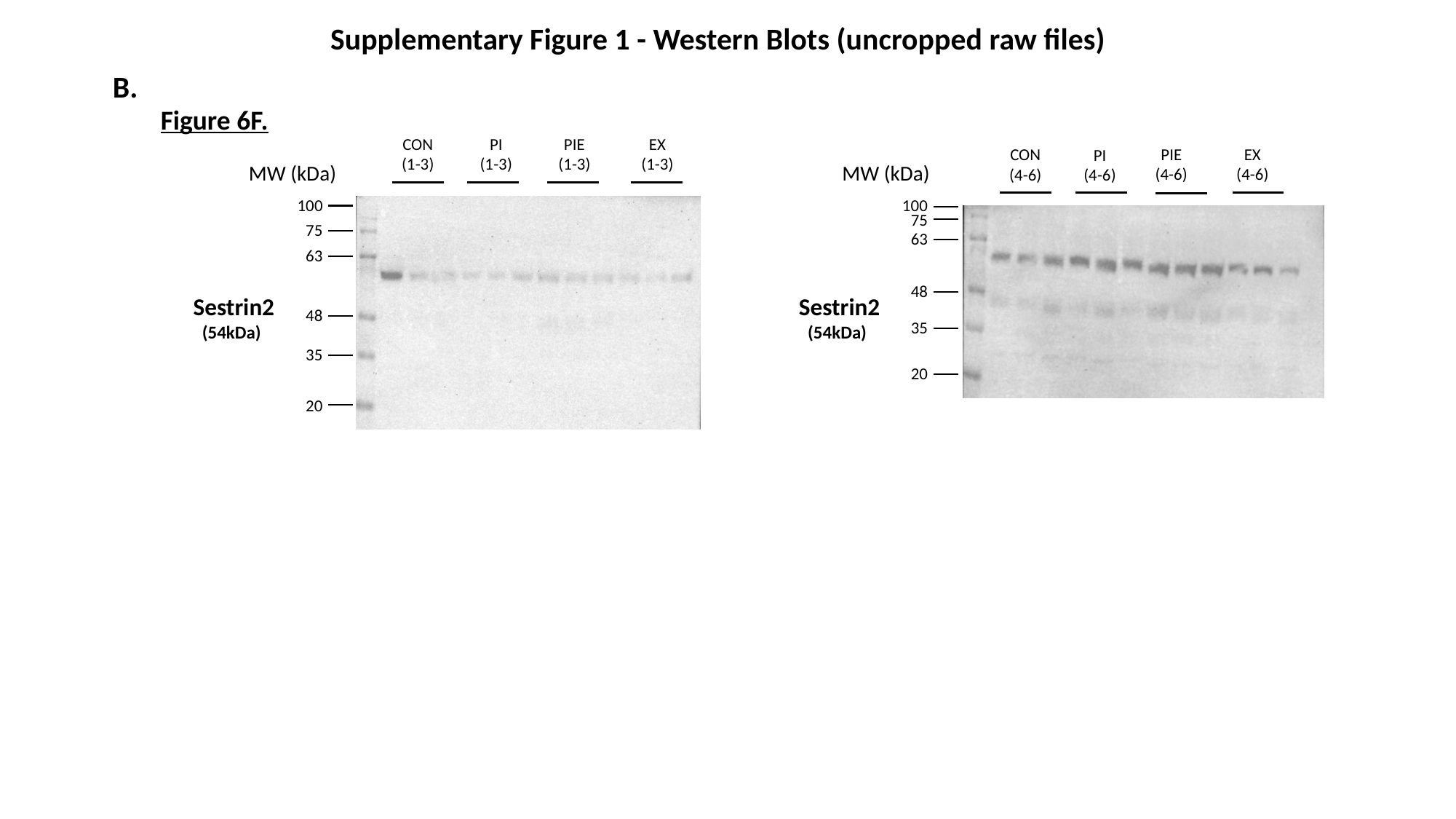

Supplementary Figure 1 - Western Blots (uncropped raw files)
B.
Figure 6F.
PI
(1-3)
PIE
(1-3)
EX
(1-3)
CON
(1-3)
EX
(4-6)
PIE
(4-6)
CON
(4-6)
PI
(4-6)
MW (kDa)
MW (kDa)
100
75
63
48
35
20
100
75
63
48
35
20
Sestrin2
(54kDa)
Sestrin2
(54kDa)

## Slide 7
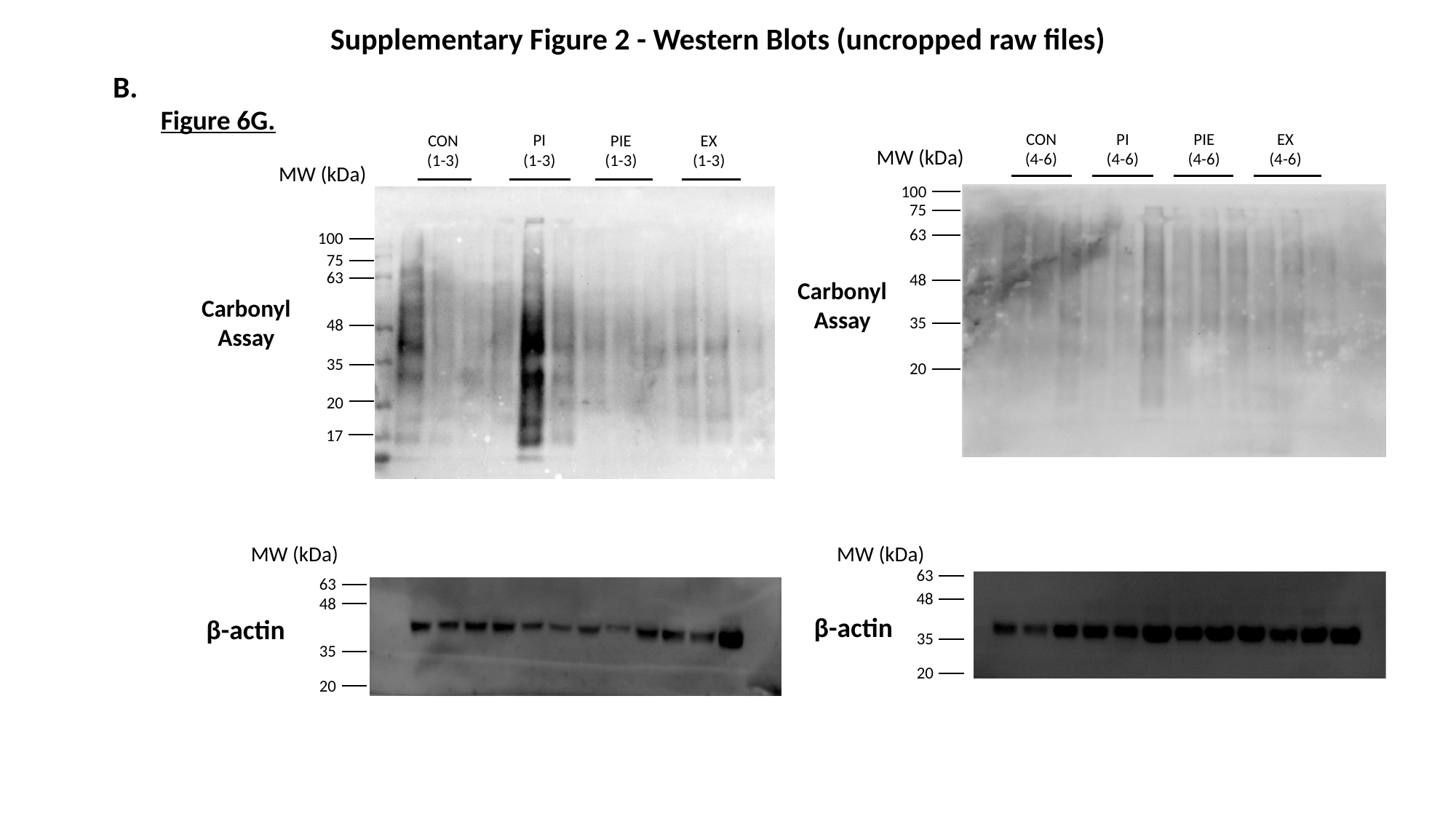

Supplementary Figure 2 - Western Blots (uncropped raw files)
B.
Figure 6G.
CON
(4-6)
PI
(4-6)
PIE
(4-6)
EX
(4-6)
PI
(1-3)
EX
(1-3)
CON
(1-3)
PIE
(1-3)
MW (kDa)
MW (kDa)
100
75
63
100
75
63
48
Carbonyl
Assay
Carbonyl
Assay
35
48
35
20
20
17
MW (kDa)
MW (kDa)
63
48
35
20
63
48
β-actin
β-actin
35
20

## Slide 8
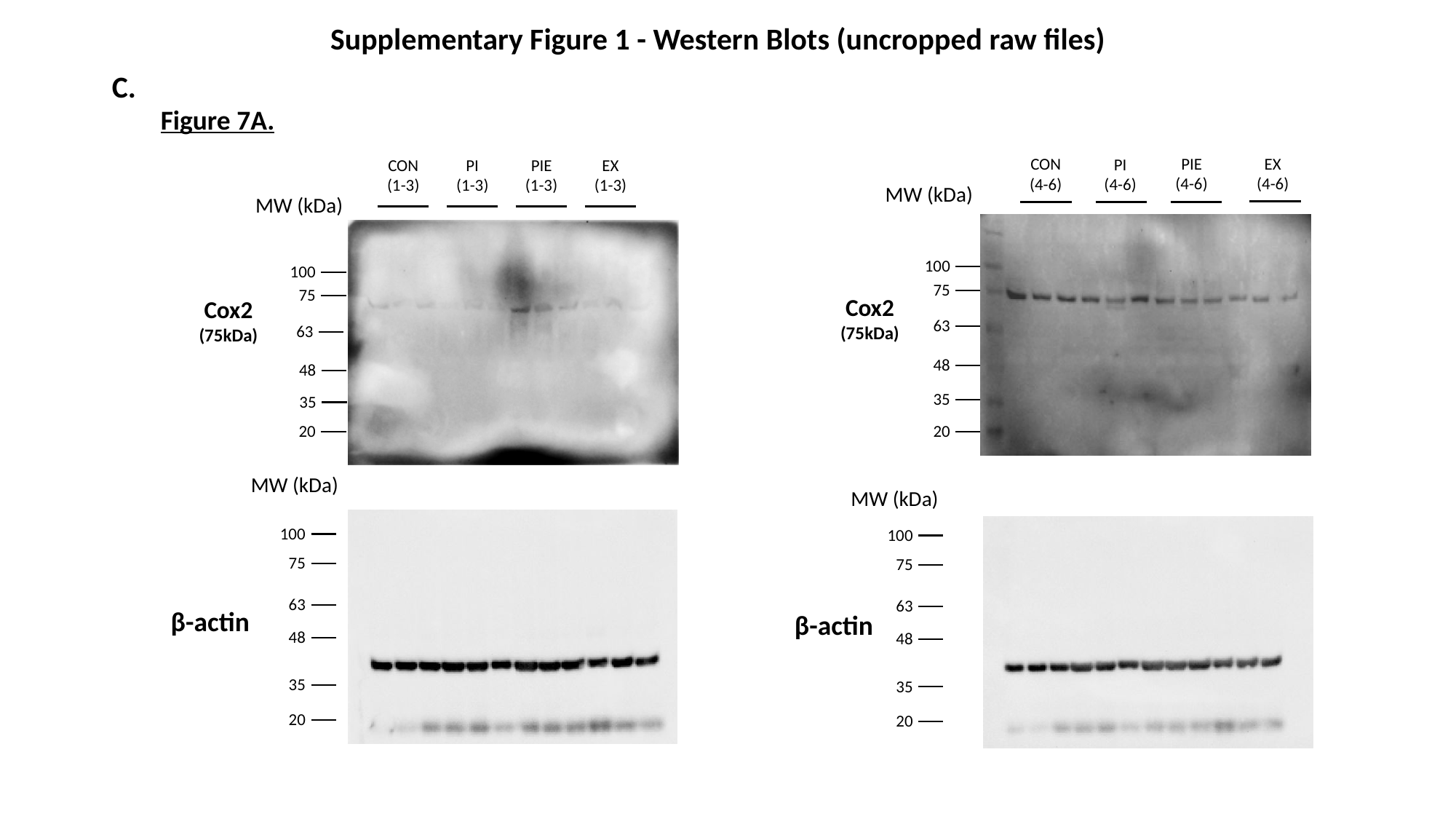

Supplementary Figure 1 - Western Blots (uncropped raw files)
C.
Figure 7A.
EX
(4-6)
PIE
(4-6)
CON
(4-6)
PI
(4-6)
CON
(1-3)
PI
(1-3)
PIE
(1-3)
EX
(1-3)
MW (kDa)
MW (kDa)
100
100
75
75
Cox2
(75kDa)
Cox2
(75kDa)
63
63
48
48
35
35
20
20
MW (kDa)
MW (kDa)
100
75
63
48
35
20
100
75
63
48
35
20
β-actin
β-actin

## Slide 9
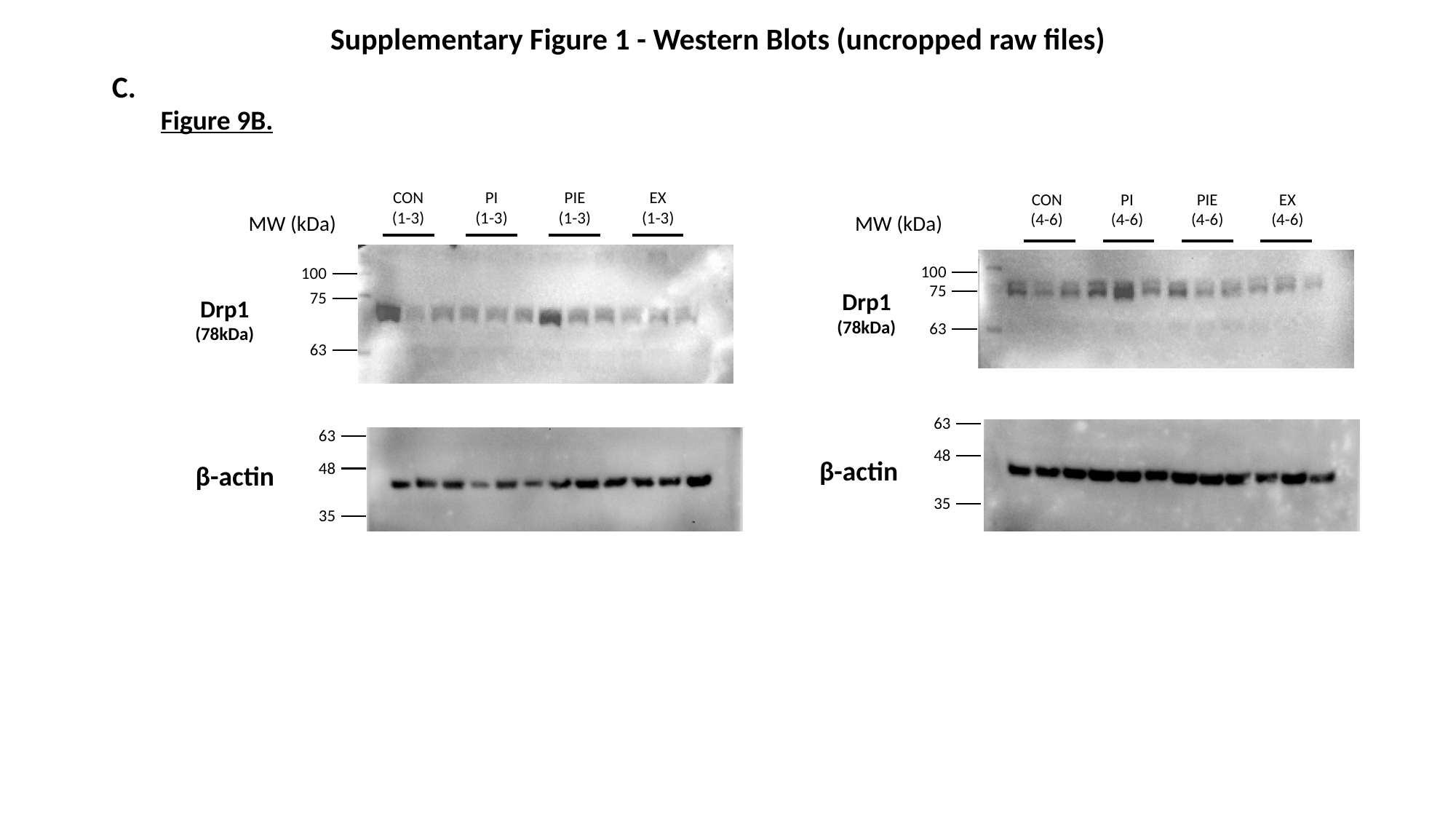

Supplementary Figure 1 - Western Blots (uncropped raw files)
C.
Figure 9B.
CON
(1-3)
PI
(1-3)
PIE
(1-3)
EX
(1-3)
CON
(4-6)
PI
(4-6)
PIE
(4-6)
EX
(4-6)
MW (kDa)
MW (kDa)
100
100
75
Drp1
(78kDa)
75
Drp1
(78kDa)
63
63
63
48
35
63
48
35
β-actin
β-actin

## Slide 10
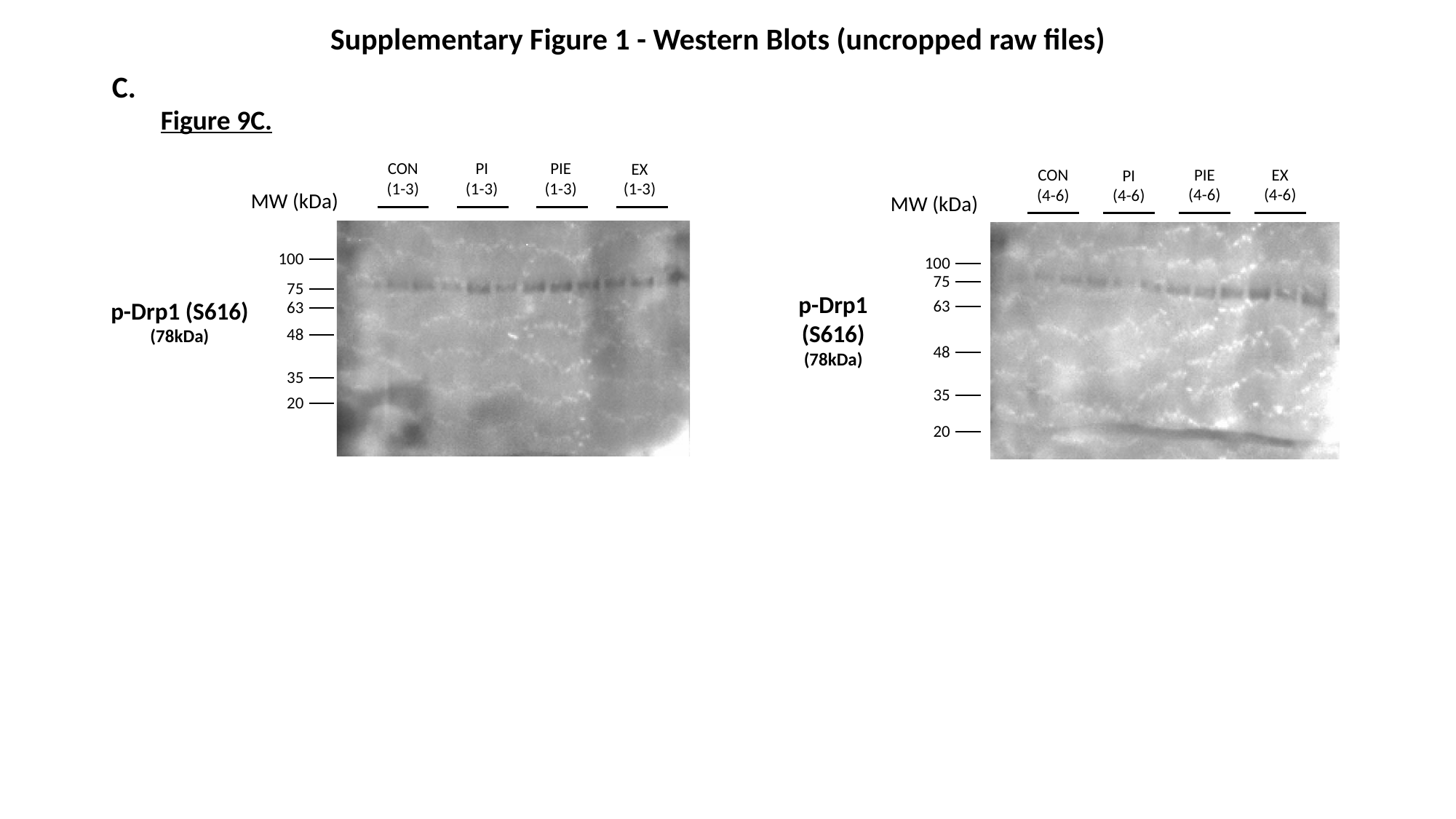

Supplementary Figure 1 - Western Blots (uncropped raw files)
C.
Figure 9C.
CON
(1-3)
PI
(1-3)
PIE
(1-3)
EX
(1-3)
PIE
(4-6)
EX
(4-6)
CON
(4-6)
PI
(4-6)
MW (kDa)
MW (kDa)
100
100
75
75
p-Drp1 (S616)
(78kDa)
p-Drp1 (S616)
(78kDa)
63
63
48
48
35
35
20
20

## Slide 11
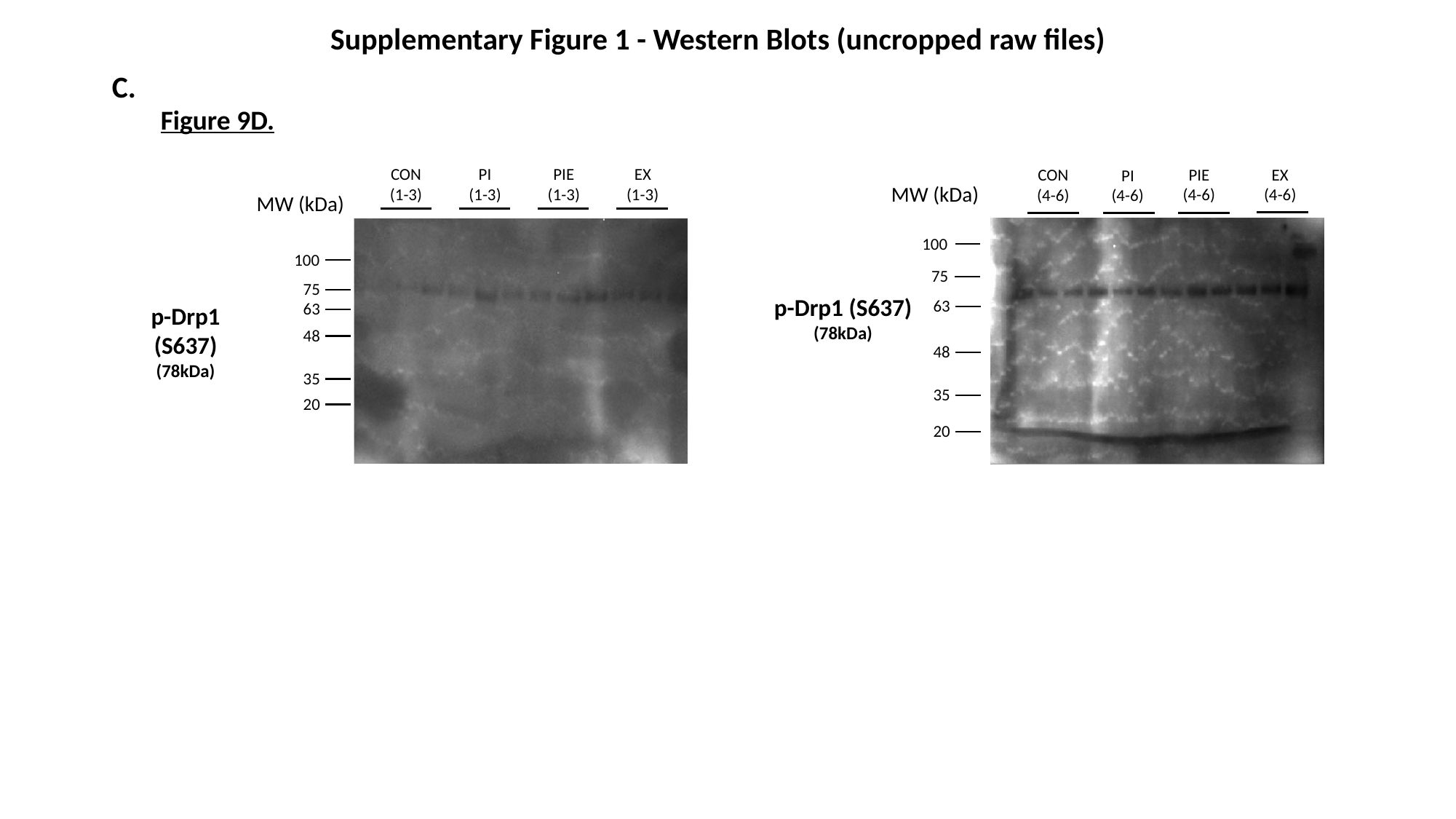

Supplementary Figure 1 - Western Blots (uncropped raw files)
C.
Figure 9D.
CON
(1-3)
PI
(1-3)
PIE
(1-3)
EX
(1-3)
EX
(4-6)
PIE
(4-6)
CON
(4-6)
PI
(4-6)
MW (kDa)
MW (kDa)
100
100
75
63
48
35
20
75
p-Drp1 (S637)
(78kDa)
63
p-Drp1 (S637)
(78kDa)
48
35
20

## Slide 12
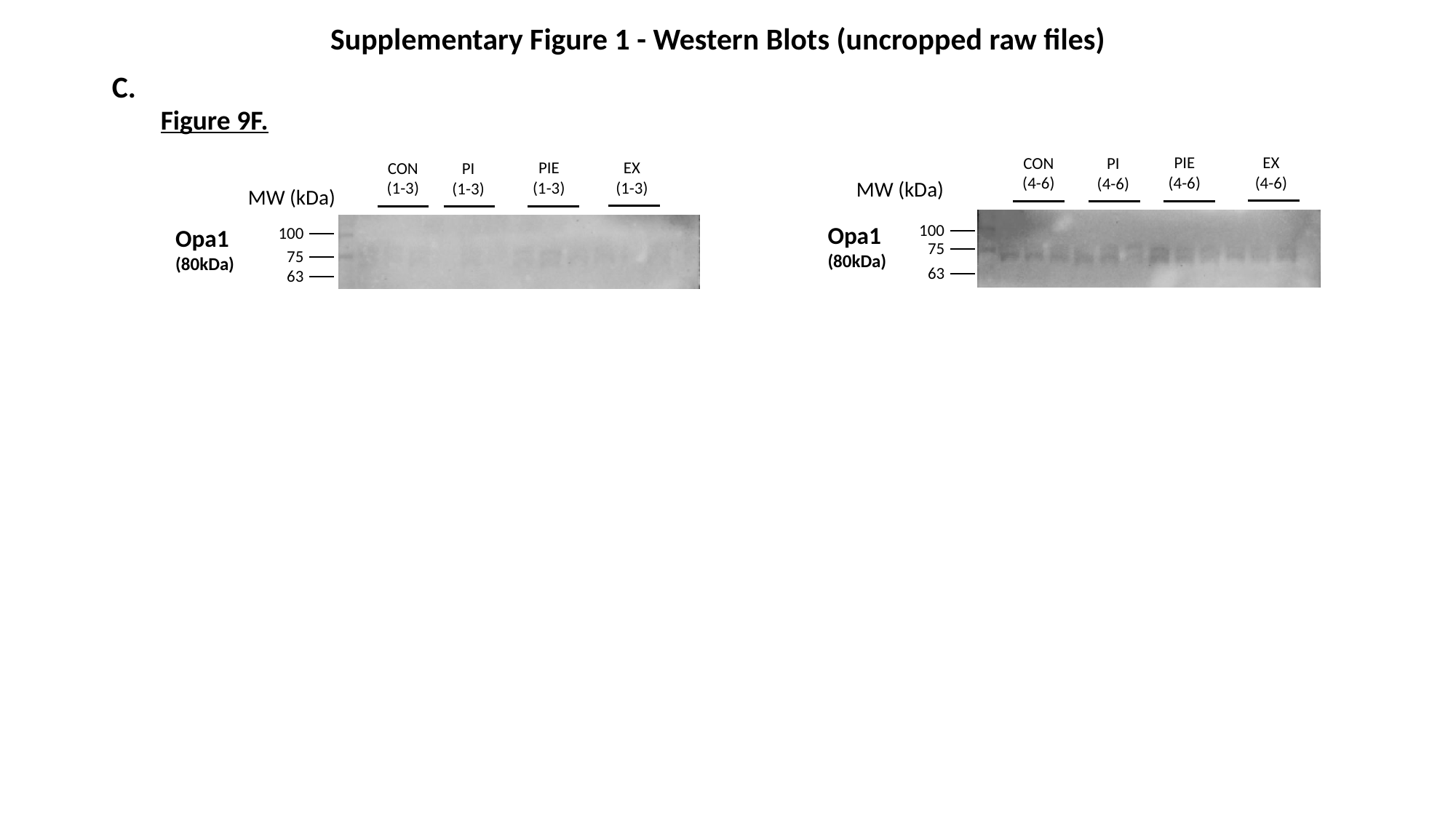

Supplementary Figure 1 - Western Blots (uncropped raw files)
C.
Figure 9F.
EX
(4-6)
PIE
(4-6)
CON
(4-6)
PI
(4-6)
EX
(1-3)
PIE
(1-3)
CON
(1-3)
PI
(1-3)
MW (kDa)
MW (kDa)
100
Opa1
(80kDa)
100
Opa1
(80kDa)
75
75
63
63

## Slide 13
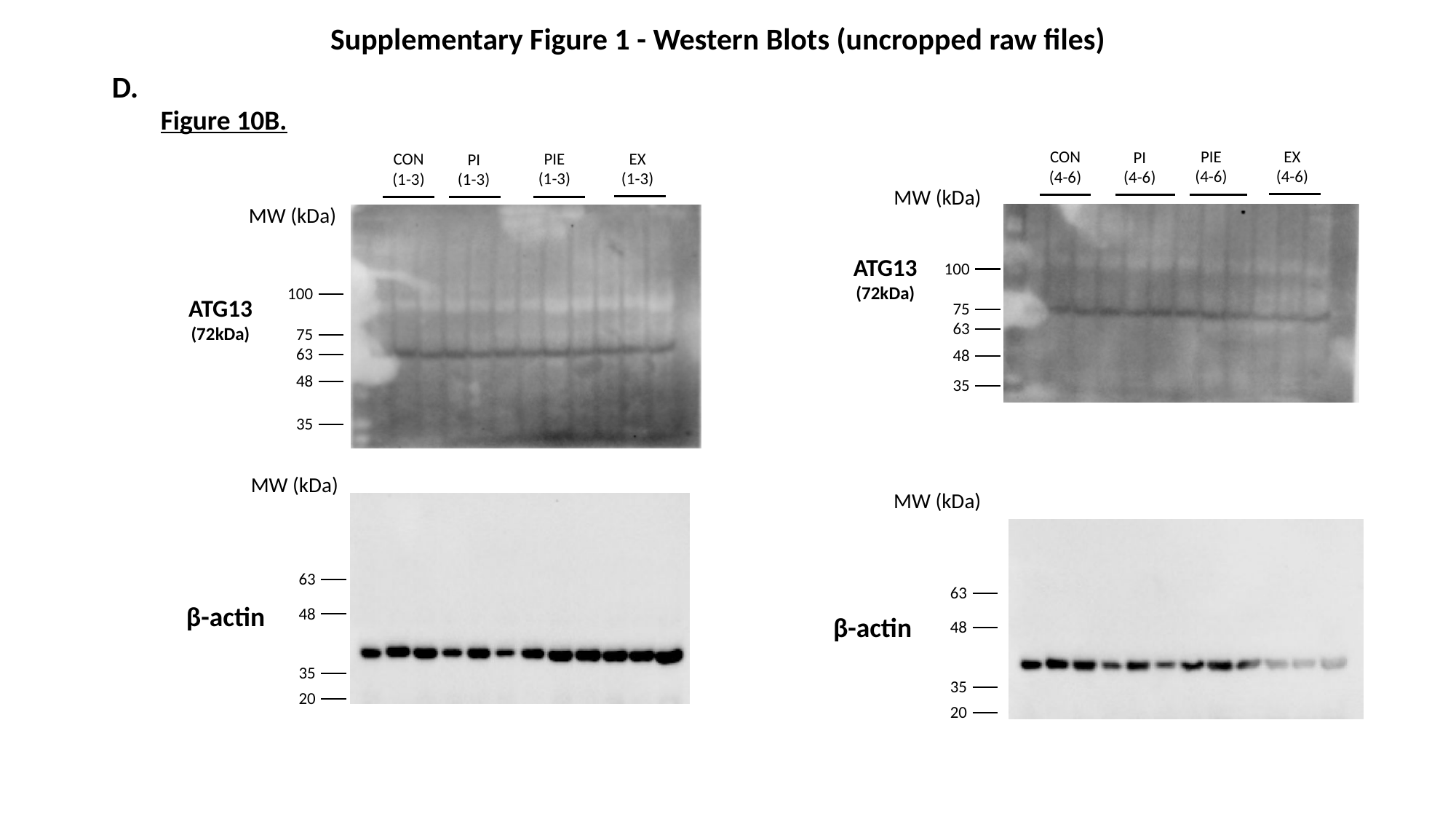

Supplementary Figure 1 - Western Blots (uncropped raw files)
D.
Figure 10B.
EX
(4-6)
PIE
(4-6)
CON
(4-6)
PI
(4-6)
EX
(1-3)
PIE
(1-3)
CON
(1-3)
PI
(1-3)
MW (kDa)
MW (kDa)
ATG13
(72kDa)
100
100
ATG13
(72kDa)
75
63
75
63
48
48
35
35
MW (kDa)
MW (kDa)
63
63
β-actin
48
β-actin
48
35
35
20
20

## Slide 14
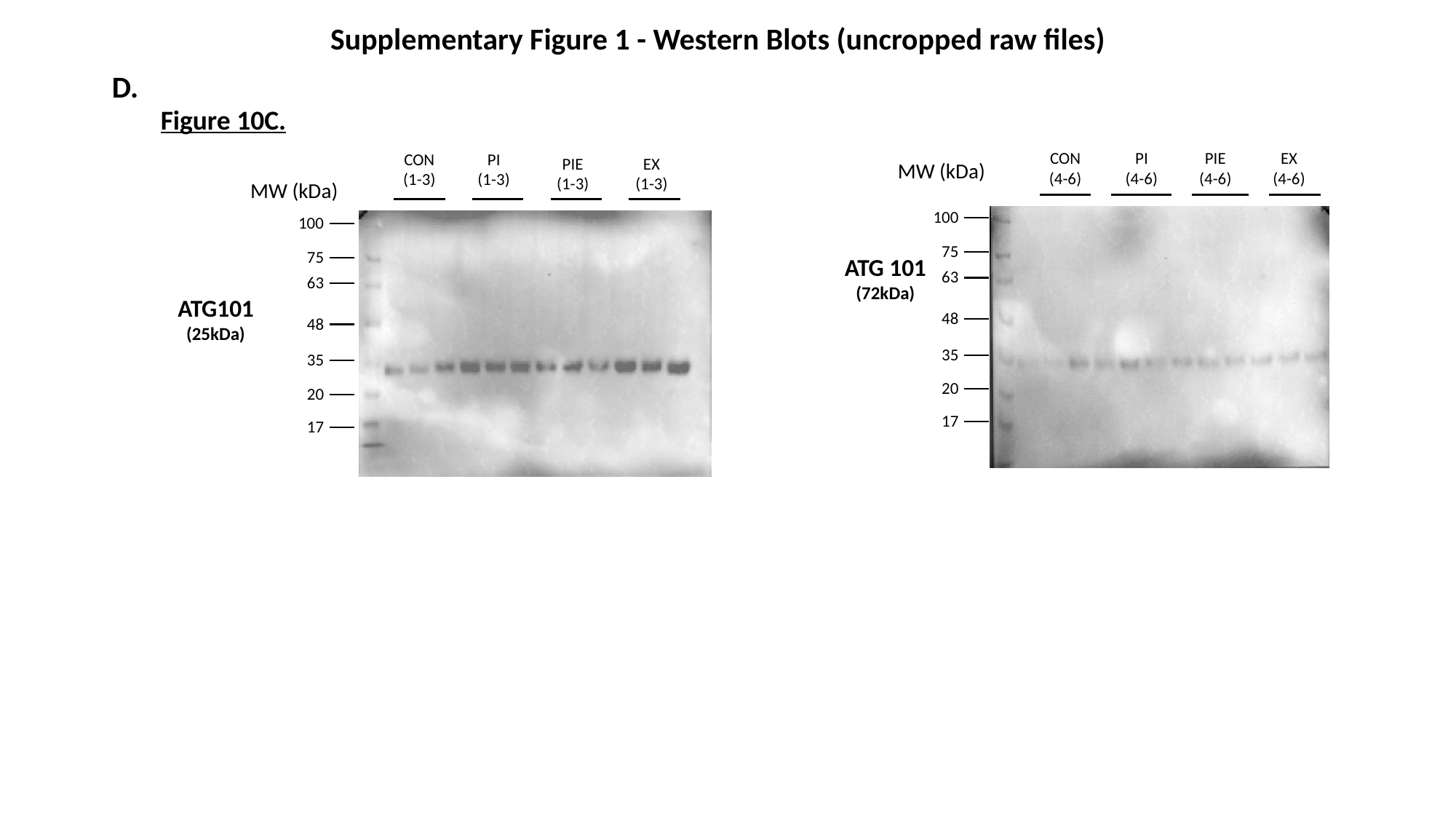

Supplementary Figure 1 - Western Blots (uncropped raw files)
D.
Figure 10C.
CON
(4-6)
PI
(4-6)
PIE
(4-6)
EX
(4-6)
PI
(1-3)
CON
(1-3)
EX
(1-3)
PIE
(1-3)
MW (kDa)
MW (kDa)
100
75
63
48
35
20
17
100
75
63
48
35
20
17
ATG 101
(72kDa)
ATG101
(25kDa)

## Slide 15
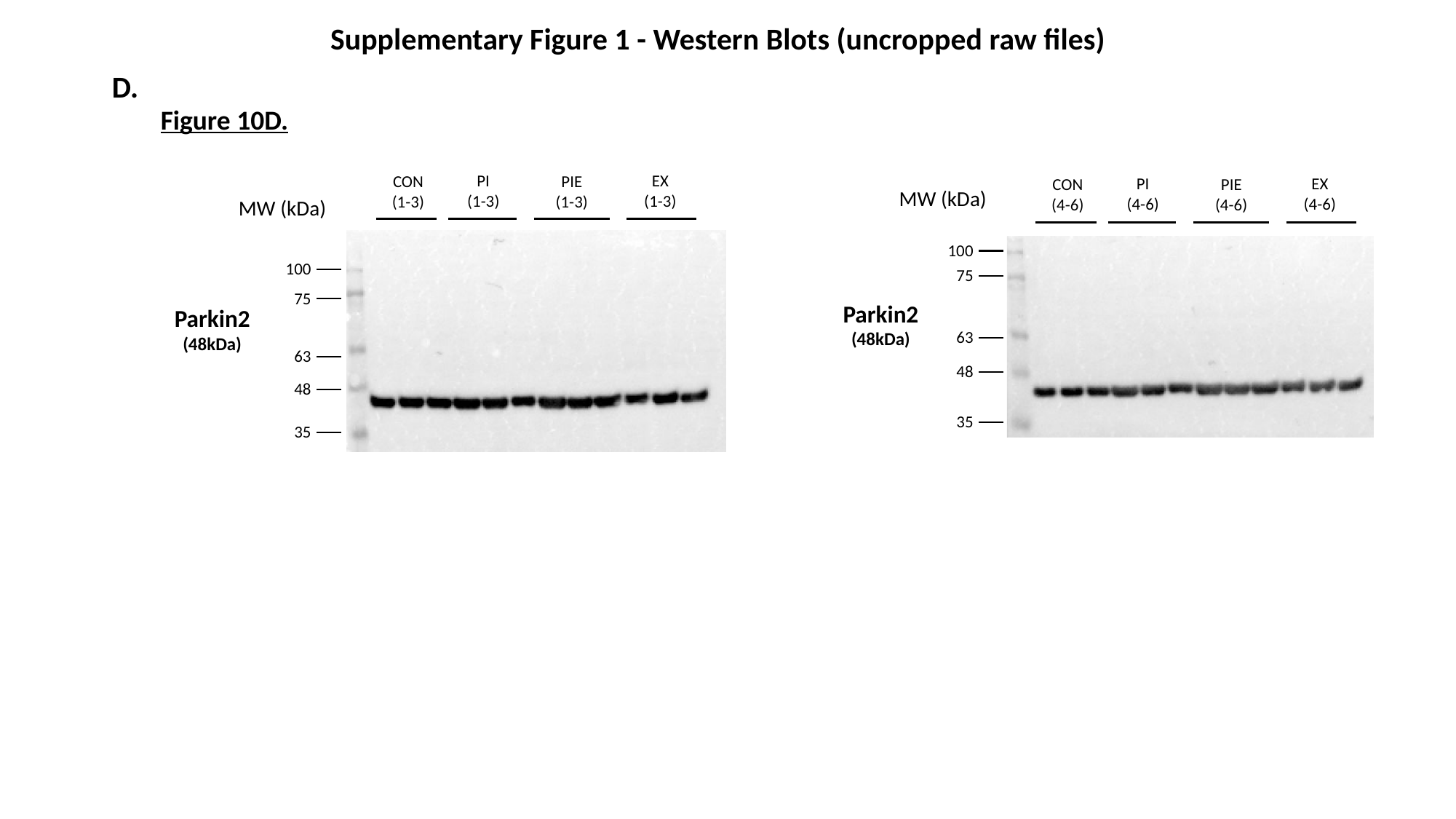

Supplementary Figure 1 - Western Blots (uncropped raw files)
D.
Figure 10D.
PI
(1-3)
EX
(1-3)
CON
(1-3)
PIE
(1-3)
PI
(4-6)
EX
(4-6)
CON
(4-6)
PIE
(4-6)
MW (kDa)
MW (kDa)
100
100
75
75
Parkin2
(48kDa)
Parkin2
(48kDa)
63
63
48
48
35
35

## Slide 16
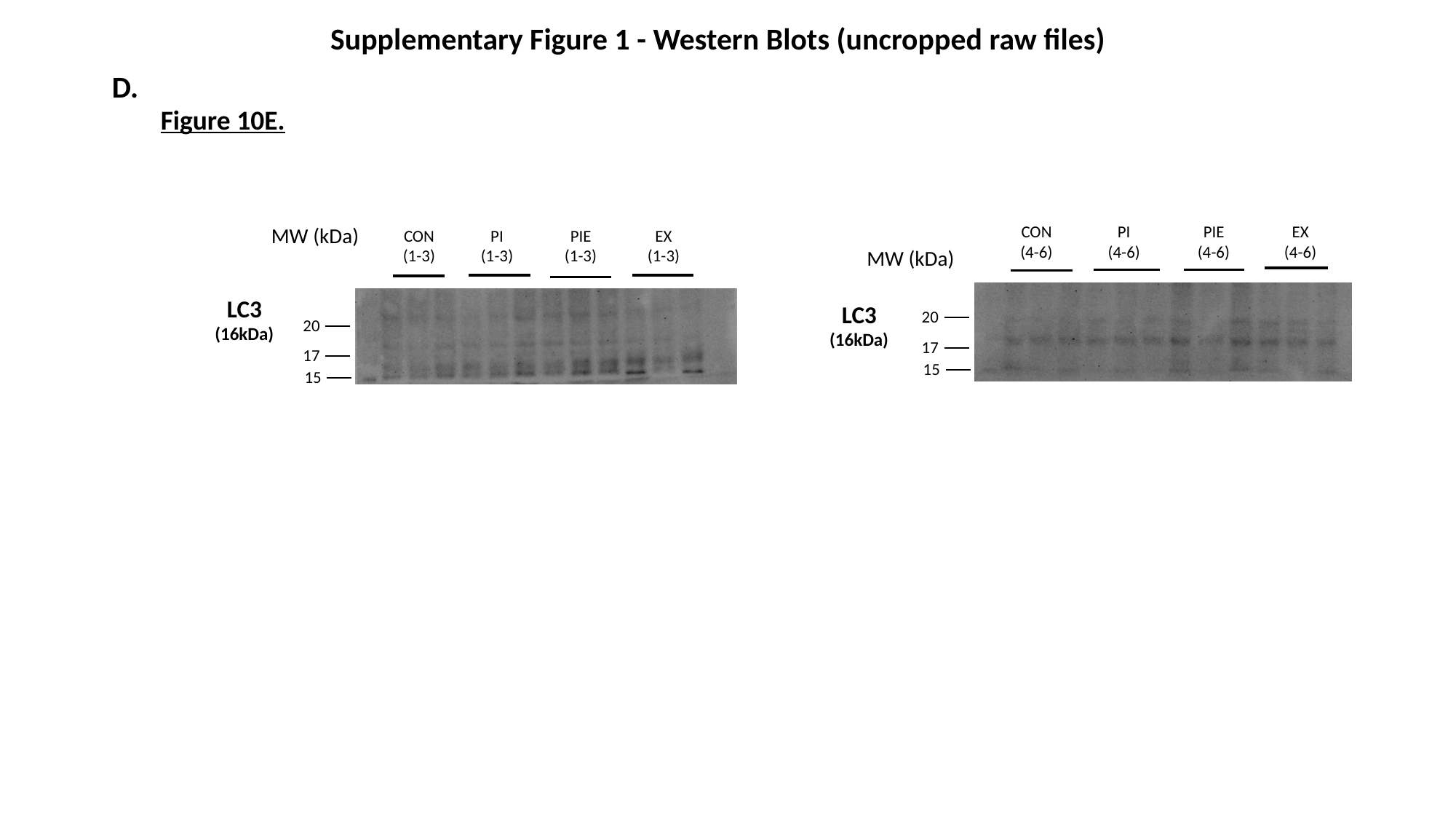

Supplementary Figure 1 - Western Blots (uncropped raw files)
D.
Figure 10E.
CON
(4-6)
PI
(4-6)
PIE
(4-6)
EX
(4-6)
MW (kDa)
CON
(1-3)
PI
(1-3)
PIE
(1-3)
EX
(1-3)
MW (kDa)
LC3
(16kDa)
LC3
(16kDa)
20
20
17
17
15
15

## Slide 17
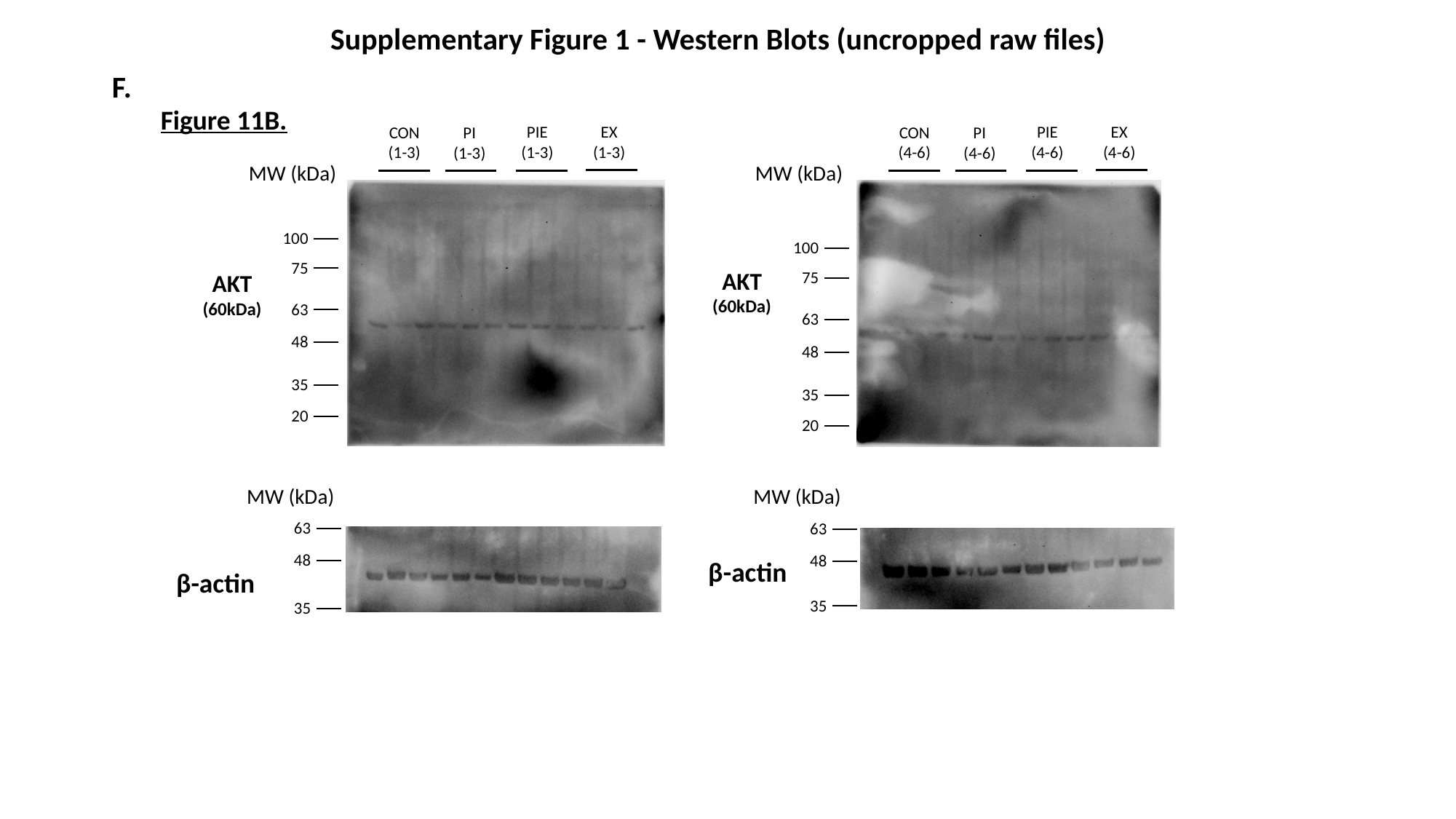

Supplementary Figure 1 - Western Blots (uncropped raw files)
F.
Figure 11B.
EX
(4-6)
EX
(1-3)
PIE
(4-6)
PIE
(1-3)
CON
(4-6)
CON
(1-3)
PI
(4-6)
PI
(1-3)
MW (kDa)
MW (kDa)
100
100
75
AKT
(60kDa)
75
AKT
(60kDa)
63
63
48
48
35
35
20
20
MW (kDa)
MW (kDa)
63
48
35
63
48
35
β-actin
β-actin

## Slide 18
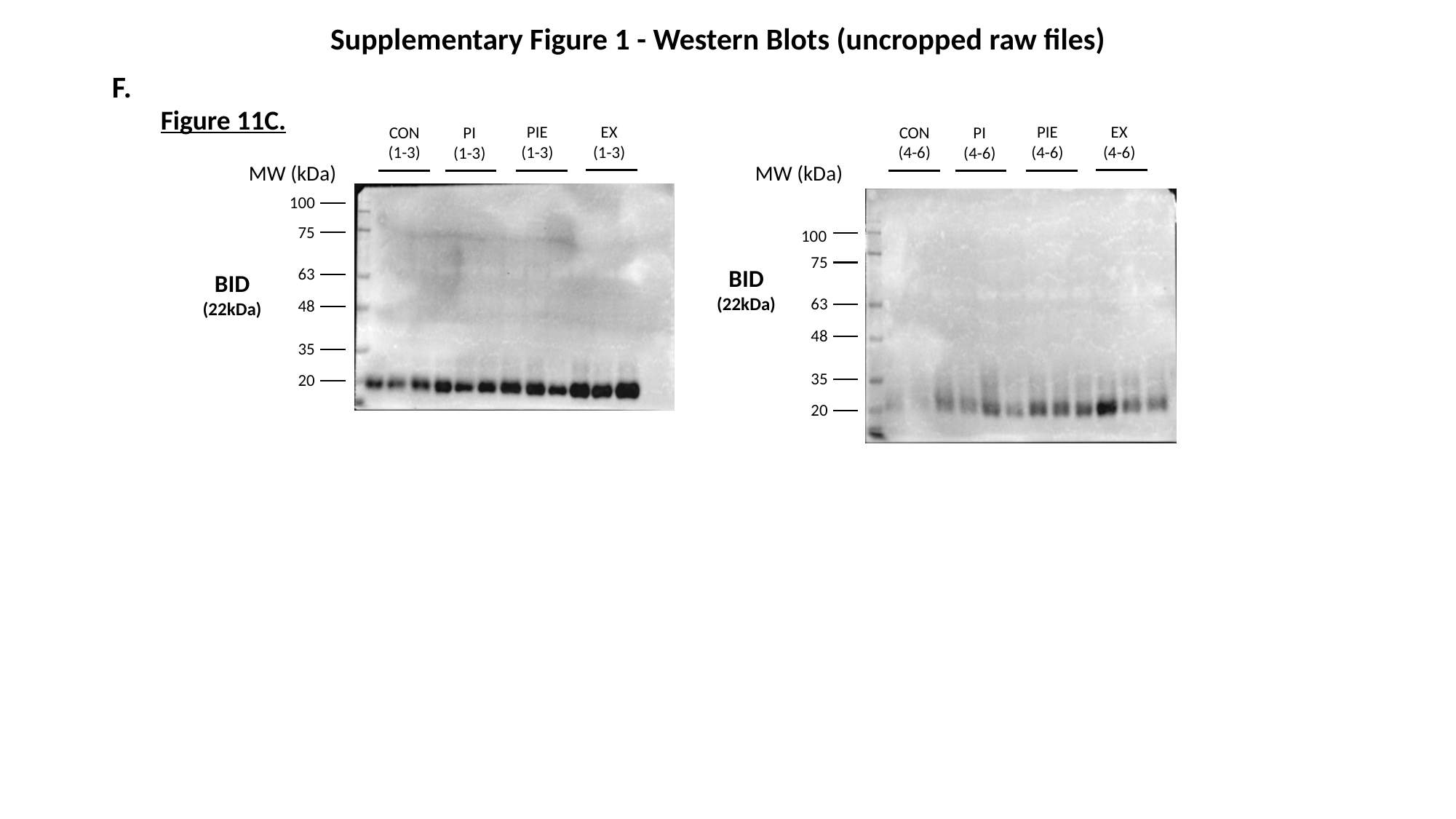

Supplementary Figure 1 - Western Blots (uncropped raw files)
F.
Figure 11C.
EX
(4-6)
EX
(1-3)
PIE
(4-6)
PIE
(1-3)
CON
(4-6)
CON
(1-3)
PI
(4-6)
PI
(1-3)
MW (kDa)
MW (kDa)
100
75
63
48
35
20
100
75
BID
(22kDa)
BID
(22kDa)
63
48
35
20

## Slide 19
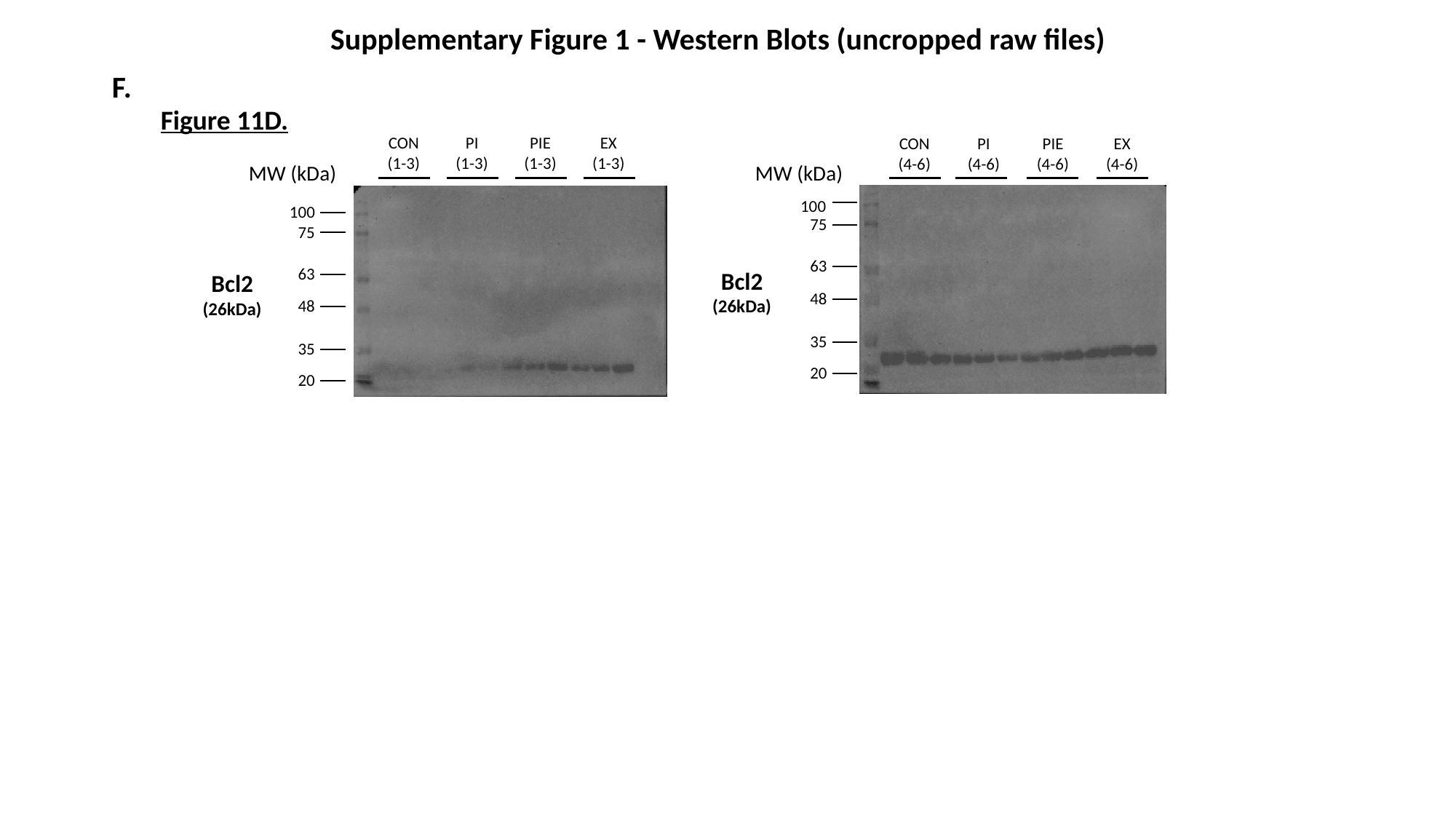

Supplementary Figure 1 - Western Blots (uncropped raw files)
F.
Figure 11D.
CON
(1-3)
PI
(1-3)
PIE
(1-3)
EX
(1-3)
CON
(4-6)
PI
(4-6)
PIE
(4-6)
EX
(4-6)
MW (kDa)
MW (kDa)
100
75
63
48
35
20
100
75
63
48
35
20
Bcl2
(26kDa)
Bcl2
(26kDa)

## Slide 20
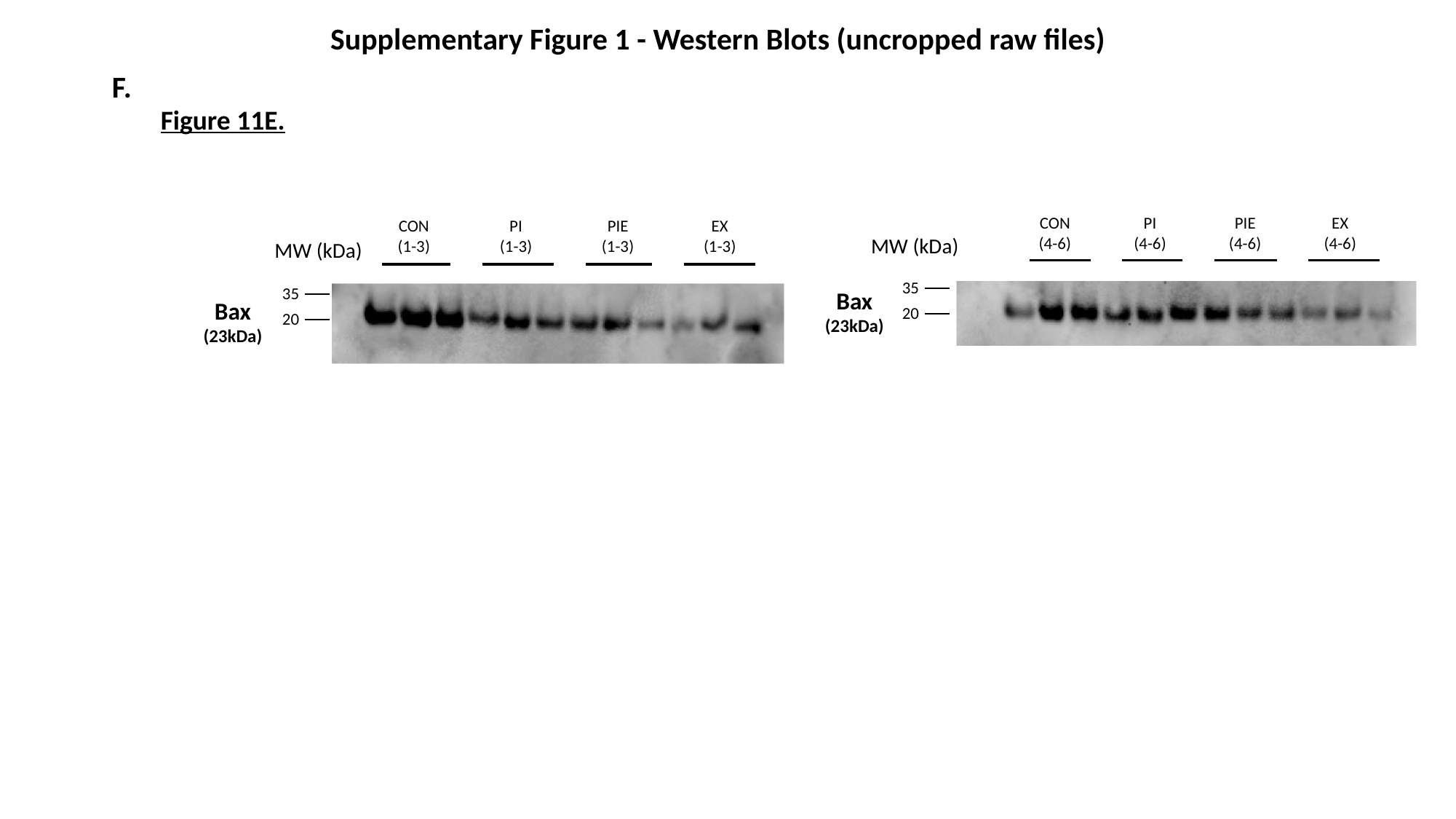

Supplementary Figure 1 - Western Blots (uncropped raw files)
F.
Figure 11E.
CON
(4-6)
PI
(4-6)
PIE
(4-6)
EX
(4-6)
CON
(1-3)
PI
(1-3)
PIE
(1-3)
EX
(1-3)
MW (kDa)
MW (kDa)
35
35
Bax
(23kDa)
Bax
(23kDa)
20
20
