# Supplementary material for: Effect of Aerobic Exercise on Oxidative Stress and Inflammatory Response During Particulate Matter Exposure in Mouse Lungs
Source: Front Physiol. 2022 Feb 3;12:773539. doi: 10.3389/fphys.2021.773539 (PMC8850364; doi:10.3389/fphys.2021.773539)
Supplement: Supplementary file 2 [file Presentation_2.PPTX]

## Slide 1
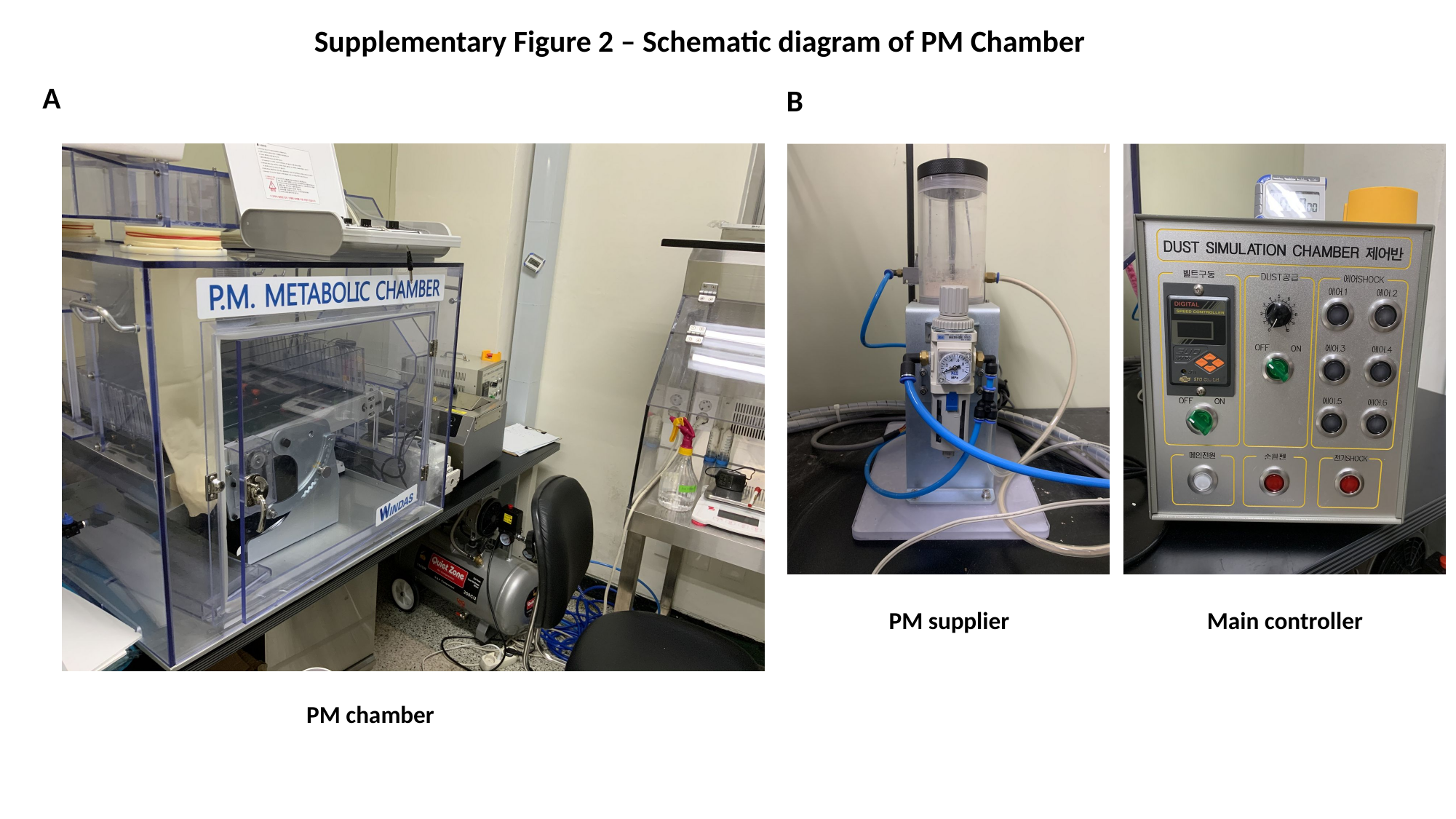

Supplementary Figure 2 – Schematic diagram of PM Chamber
A
B
PM supplier
Main controller
PM chamber

## Slide 2
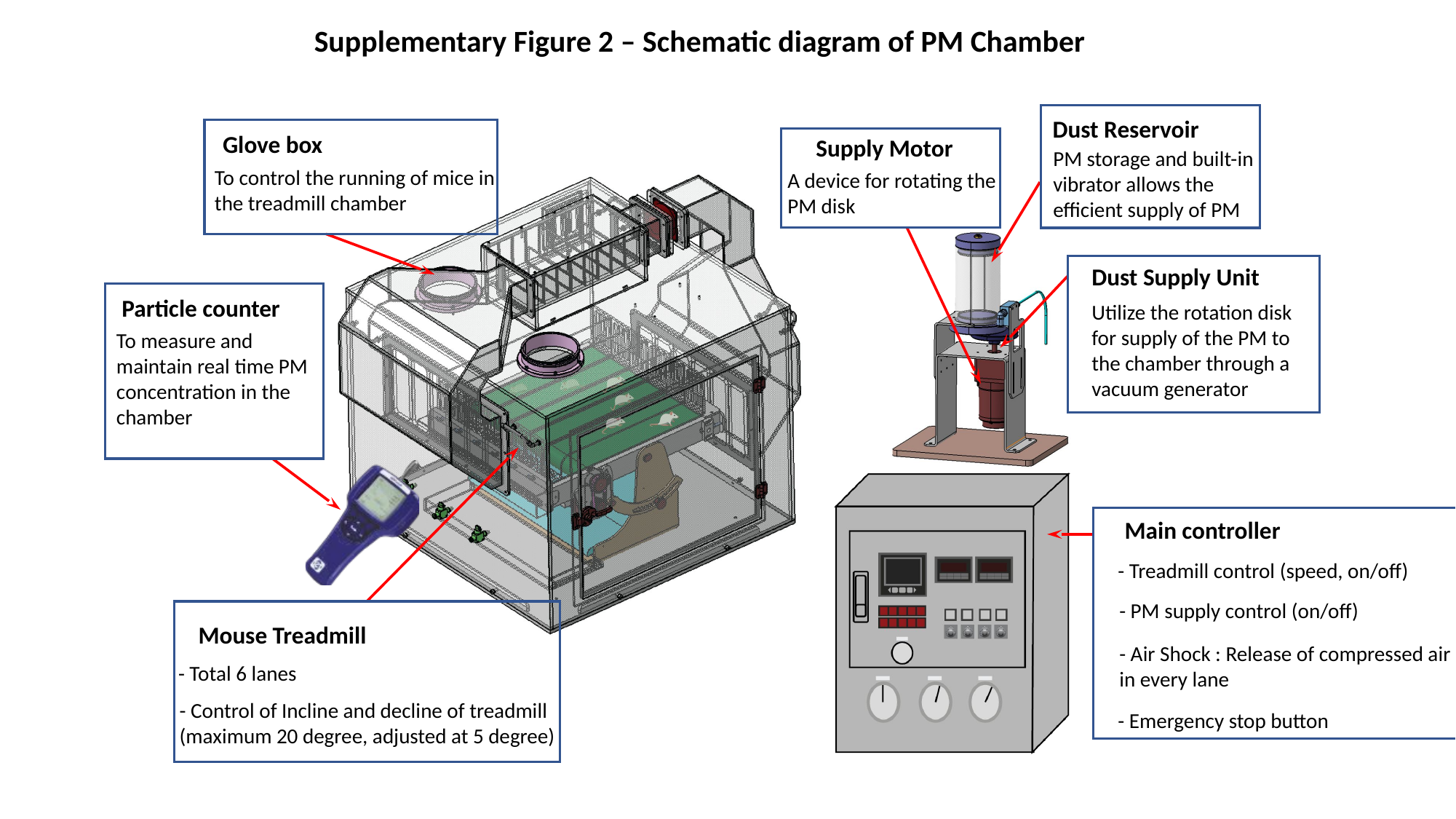

Supplementary Figure 2 – Schematic diagram of PM Chamber
Dust Reservoir
Supply Motor
PM storage and built-in vibrator allows the efficient supply of PM
A device for rotating the PM disk
Dust Supply Unit
Utilize the rotation disk for supply of the PM to the chamber through a vacuum generator
Main controller
- Treadmill control (speed, on/off)
- PM supply control (on/off)
- Air Shock : Release of compressed air in every lane
- Emergency stop button
Glove box
To control the running of mice in the treadmill chamber
Mouse Treadmill
- Total 6 lanes
- Control of Incline and decline of treadmill
(maximum 20 degree, adjusted at 5 degree)
Particle counter
To measure and maintain real time PM concentration in the chamber
